# Oncolytic Viruses: An Inventory of Shedding Data from Clinical Trials and Elements for the Environmental Risk Assessment

**DOI:** 10.3390/vaccines11091448

**Published:** 2023-09-01

**Authors:** Sheela Onnockx, Aline Baldo, Katia Pauwels

**Affiliations:** Sciensano, Service Biosafety and Biotechnology, Rue Juliette Wytsmanstraat 14, B-1050 Brussels, Belgium; aline.baldo@sciensano.be (A.B.); katia.pauwels@sciensano.be (K.P.)

**Keywords:** oncolytic virus, shedding, environmental risk assessment, biosafety, clinical trials, cancer

## Abstract

Attenuated and/or genetically modified oncolytic viruses (OV) gain increasing interest as a promising approach for cancer therapy. Beside the assessment of subject safety, quality and efficacy aspects of medicinal products for human use, genetically modified viruses are also governed by EU regulatory frameworks requiring an environmental risk assessment (ERA). An important element to be assessed as part of the ERA is the incidence of exposure to OV of individuals, other than the trial subjects, and the environment. The evidence-based evaluation of shedding data is considered to be decisive in that context, as it may impact the OV capacity to be transmitted. This is particularly true for OV still able to (conditionally) replicate as opposed to replication-defective viral vectors commonly used in gene therapy or vaccination. To our knowledge, this article presents the most extensive and up-to-date review of shedding data reported with OV employed in clinics. Besides the identification of a topical need for improving the collection of shedding data, this article aims at providing an aid to the design of an appropriate shedding study, thereby relying on and further complementing principles described in existing guidelines issued by European and international institutions.

## 1. Introduction

**Figure 1:**
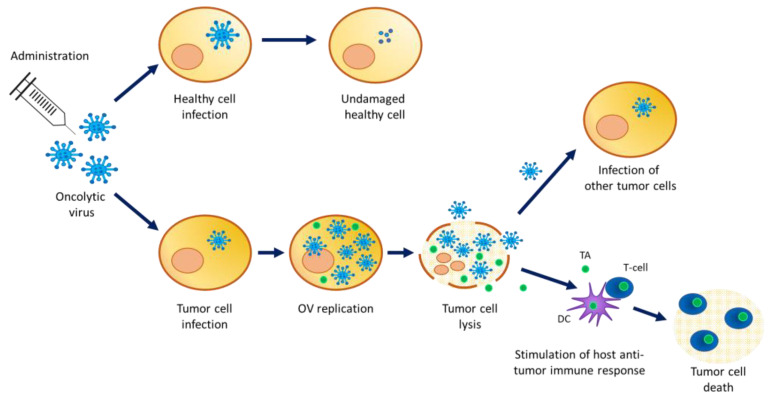
Mechanism of action of oncolytic viruses. OV infect healthy cells but cannot replicate. High infection of cancer cells where OV replicate and package for the production of new viral particles. A high viral load inside the cell causes tumor cell lysis releasing viral particles and tumor antigens in the cancer microenvironment. The viral particles’ progeny can infect other tumor cells while the released tumor antigens stimulate the host anti-tumor immune response. TA = tumor antigen; DC = dendritic cell.

Oncolytic viruses (OV) are (conditionally) replication competent viruses (with low pathogenicity) that are designed to be able to selectively replicate in tumor cells, leading to their destruction by the direct lysis of host tumor cells, while sparing normal cells. Accumulating evidence in oncovirotherapy demonstrates that OV infection can also trigger specific antitumor immune effects. Cellular proteins released from OV-lysed tumor cells elicit an interaction with the innate immune system through the activation of dendritic cells, which in turn stimulate adaptive immunity (Figure 1).

**Figure 2:**
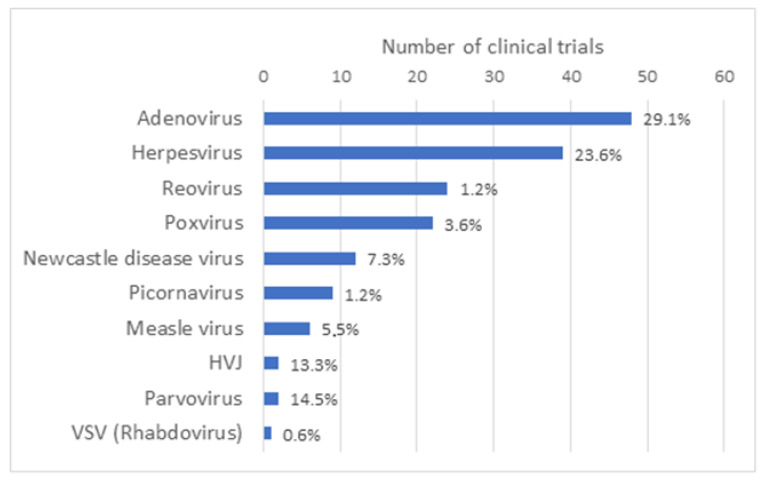
Type of oncolytic viruses used in oncolytic virus clinical trials. Based on the results obtained in the Supplementary Table S1 of this article, a graph representing the number of clinical trials that have been performed by type of oncolytic virus all over the world has been generated. Taken together, the number of clinical trials with Adenovirus and Herpesvirus represents slightly more than half of all clinical trials with OV.

OV can be naturally occurring viruses that have natural tropism to neoplastic cells, such as Reoviruses [1], Newcastle disease virus (NDV) [2] and Vesicular stomatitis virus (VSV) [3]. OV can also be laboratory-adapted attenuated virus strains, such as attenuated Measles viruses, which acquired the ability to use, for viral entry, receptors that are overexpressed on the surface of malignant cells [4]. Some OV have also been genetically modified (GM) to enhance their antitumor specificity, safety and immunogenicity, for example by delivering immuno-stimulatory agents or therapeutic agents or by triggering novel cancer-specific acquired immune responses against tumor antigens. As reported by Madeco et al., in 2020, approximately two-thirds of the studies involving OV use GM viruses [5]. The growing list of virus platforms applied as oncolytic virotherapy or even as oncolytic immunotherapy illustrates the increasing clinical interest in OV as effective cancer therapeutics, either as a single-agent therapeutics or in combination with chemotherapy, radiation treatment or immune-based therapeutic regimens. OV under clinical investigation include Adenovirus (Ad) (48), Herpesvirus (39), Reovirus (24) and Poxvirus (vacciniavirus 21/22; fowlpox virus 1/22), along with other candidate viruses, such as Measles virus, Gamma-Herpes virus, Parvovirus, Retrovirus and VSV that have been reported in single or a limited number of clinical trials (Figure 2). Characteristics of the most widely used OV such as their structure, virion size and receptor usage, as well as some of the main advantages and disadvantages, have been reviewed elsewhere [6].

While the approval in the US and Europe in 2015 [7,8,9] (and later also elsewhere) of Imlygic^®®^ (also known as talimogene laherparepvec, T-VEC and OncoVex), an oncolytic Herpes Simplex Virus (HSV), for the treatment of advanced melanoma can be considered as a milestone in the development of OV therapy, it should be noticed that three other OV have been approved for commercialization. A wild-type Enteric Cytopathogenic Human Orphan type 7 (ECHO-7) Picornavirus, with trade name Rigvir^®®^, was first approved in 2004 in Latvia for the treatment of melanoma [10]. Earlier in 2019, the distribution of this medicinal product was stopped due to manufacturing issues and finally suspended for marketing authorization in mid-2019 [11]. A GM Adenovirus called Oncorine (H101) was approved in 2005 in China for subjects with head and neck cancer [12]. More recently, in June 2021, a GM HSV known as Teserpaturev/G47Δ (Delytact^®®^) received conditional approval in Japan for the treatment of glioblastoma [13]. Meanwhile, a number of OV have also reached an advanced stage of clinical development and are used in phase III clinical trials, such as a Newcastle disease virus to treat colorectal cancer [14,15], a vaccinia virus called Pexa-Vec (formerly JX-594) for the treatment of hepatocellular cancer [16], a Reovirus, Reolysin, as part of a combination therapy for the treatment of squamous cell carcinoma of the head and neck [17], and CG0070 Adenovirus for the treatment of non-muscle-invasive bladder cancer [18].

The clinical development of investigational products is subject to strict regulatory requirements concerning, in particular, quality, efficacy and safety aspects. In the case of GM viruses, these requirements not only pertain to the safety of the subject itself, but also to the safety of human health at large and the environment. The identification and evaluation of potential adverse effects on human health and the environment when directly exposed to the GM viruses are performed within a dedicated environmental risk assessment (ERA) as part of the regulatory application for clinical trials or marketing applications of medicinal products containing genetically modified organisms (GMO).

The evaluation of exposure pathways through which GM viruses and viral vectors with their inserted DNA sequence may interact with humans (other than subjects receiving treatment with the viral vector), or the environment, is of major importance in the ERA. Pathways of exposure include leakage upon administration (spreading) of the investigational product, accidental release during its administration, and shedding of the product by the trial subject. Shedding corresponds to the dissemination of viral (vector) particles in any form into the environment via excreta (feces, secreta (e.g., urine, sweat, saliva, nasopharyngeal fluids, lacrimal fluid, semen)), skin (wounds, pustules, sores) and blood from the treated subject [19]. Unlike gene therapy with recombinant viruses or viral vectors that are rendered replication-defective, oncovirotherapy is based on the (conditional) replication competency of the OV. As a consequence, the shedding of OV may be compared to replication-deficient viral vectors. Collecting information on whether and how OV may be released into the environment from subjects is therefore a critical step in the ERA. In addition, the likelihood of the exposure of personnel handling the vaccine or involved in clinical care (occupational exposure), close contacts of the subjects (living under the same roof or caregiver at the subject’s home) and the environment (including animals, plants and micro-organisms) necessitates an evaluation of several aspects, including the environmental stability of the OV, the person-to-person or person-to-animal transmissibility of the OV, and their capacity to exchange genetic information with circulating viruses.

While several guidelines are recommending to examine shedding as early as possible in the clinical development (see hereunder), in practice, rather limited attention is given to shedding data compared to the relatively extensive collection and assessment of data supporting the safety, quality and efficacy assessment of the OV.

This paper presents a review of shedding data collected during the clinical development of OV and aims at providing an aid in the design of an appropriate shedding study.

## 2. Materials and Methods

### Literature Review

Based on general reviews on OV recently published in PubMed [5,20], a first list of manuscripts containing results of clinical trial data using an OV was created. This list was completed by a literature review in the PubMed database on 8 June 2023. The search for the keywords “oncolytic virus therapy” was filtered for “clinical trials” and “randomized controlled trials”. In this second list, only manuscripts written in English and containing reports of clinical trial data using an OV were kept. Reports of preclinical data, clinical protocols without data, review manuscripts and retrospective studies were rejected from the list. Both lists were put together and different manuscripts related to the same clinical trial were combined as a single entry in a final list.

This final list counts 165 manuscripts representing 165 different clinical trials and each trial was evaluated for multiple variables. Variables assessed included the phase of the clinical trial, the number of subjects treated, the type of virus used, the nature of the viral backbone (i.e., native virus, modified or recombinant virus), the transgene, the type of cancer treated, the use of single-agent or combination regimens and viral genome shedding data. With respect to the viral genome shedding, the authors assessed for each of the studies whether viral genome shedding was assessed and reported, and more particularly, which body tissues and/or fluids were assessed for viral particles, which detection test was used, whether viral particles were detected and for how long, and whether shed particles contained infectious viral particles. This final list of 165 manuscripts can be found in online Supplementary Table S1.

## 3. Environmental Risk Assessment

In addition to the regulatory requirements common to all (investigational) medicinal products, the use of GM viruses for pre-clinical investigation, clinical trials as well as their marketing as medicinal product is covered by the legislation regulating the use of GMO, which encompasses an ERA.

The ERA relies on a well-defined methodology and should be conducted on a case-by-case basis. It starts with the identification and characterization of potential hazards associated with the GMO on human health, with focus on individuals other than subjects or those vaccinated, and on the environment at large, including animals, plants and microorganisms. Concurrently and as part of a second risk component, the probability of occurrence of potential hazards under the conditions of use is assessed. Both components led to an estimation of the risk to human health and the environment posed by each identified hazard of the GMO by combining the probability of its occurrence and the magnitude of its consequences. An overall risk is then determined by combining all of the individual risks [21,22]. The ERA is based on a weight of evidence methodology encompassing both qualitative and quantitative considerations, and is described using qualitative terms ranging from high to moderate, low and negligible [23]. After overall risk determination, it is examined whether risk management measures need to be implemented in order to minimize the likelihood of adverse effects occurring. If no risks are identified that require management, no risk management strategy need be defined.

Numerous viruses have been used to design and develop OV therapy. The identification of potential hazards, the first step in the ERA, should take into account the characteristics of the OV and the properties of the inserted gene sequences and the gene products. This implies that due consideration of aspects such as the extent of attenuation, the replication capacity, tropism, biodistribution, genetic stability and the capacity to recombine with other wild-type viruses should be evaluated on a case-by-case basis. The properties of the parental viruses from which OV are derived may provide valuable starting information, taking into account that, in general, OV developed for therapeutic application are less virulent or infectious.

The development of different oncolytic viral systems for cancer therapeutic applications relies to a major extent on the genetic modification of viruses. Several strategies to enhance the therapeutic effect of oncolytic virotherapy involve the genetic “arming” of replicating viruses with transgenes, such as tumor suppressor genes, immune regulatory genes, apoptosis inducing genes, angiogenesis inhibitory genes and genes coding for pro-drug converting enzymes or heat shock proteins. Besides the insertion of transgenes with an inherent therapeutic effect, other sequences can be inserted or deleted that are involved in the targeting of OV [24].

Inserted gene sequences and their gene products should be considered for their potential impact on the viral life cycle alterations (e.g., viral tropism, entry, transcription/translation, replication, transmission), on the capacity of recombination of the virus or on the host (e.g., immune modulation, pathogenesis). All of these elements may alter biodistribution and persistence in the subjects and may impact shedding following administration of the OV.

In the next section, we will focus on elements that may have an impact on the shedding properties of OV, based on examples in the clinical field, and elaborate on the assessment of shedding data as one of the key aspects in the outcome of the ERA of OV.

## 4. Shedding Analysis

### 4.1. Definition

As mentioned above, shedding corresponds to the dissemination of viral (vector) particles in any form into the environment via the excreta or secreta, skin and blood from the treated subject [19]. While indirectly related, the evaluation of the shedding pattern of OV addresses issues that are distinct from biodistribution, because the latter focuses on dissemination and persistence within the host tissues, thereby mainly impacting the subject, while shedding can be considered as one of the main pathways through which GM OV may come into contact with individuals other than the treated subject. Another consideration to be made for the purpose of this article is that blood and related products, like peripheral blood mononuclear cells, serum or plasma, are not considered as biological fluids that can spontaneously be shed into the environment. However, blood and related products could be a source of exposure for personnel in clinical settings (e.g., during intravenous administration of the product, etc.) or for close contacts of the trial subjects (e.g., direct contact with open wounds). Addressing the release of OV through secretions and/or excreta of the subject is a key element to be performed during the ERA of the clinical use of OV and should be examined as early as possible in the clinical process.

### 4.2. Detection

**Figure 3:**
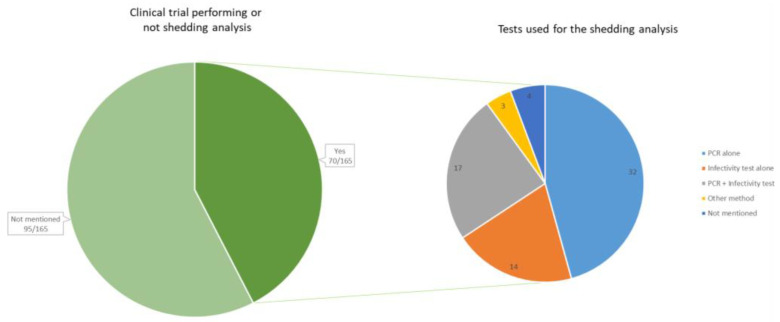
On the left, the number of clinical trials that reported information on viral genome shedding (*n* = 70). On the right, the method of detection used during the shedding analyses, reported within the 70 clinical trials. The method “PCR” includes all types of PCR (quantitative PCR, real-time PCR, reverse transcription PCR, etc.). The infectivity test includes cell culture and plaque assays. Other methods include viruria and direct fluorescence hexon protein assay.

From our review of the literature (see Supplementary Table S1), it is observed that the results of shedding analysis are available in about half of the early phases of the OV development. Shedding analysis and results were reported in 70 clinical trials (42.4%) (all phases confounded), while it was not specified whether shedding analysis was conducted in 95 clinical trials (57.6%) (Figure 3).

The test method used to assess the shedding potential of oncolytic viruses should be sufficiently sensitive [25,26,27]. Polymerase chain reaction (PCR) and infectivity are mainly used for the detection of shed virus/vector. A quantitative PCR assay to detect viral genetic material is recommended to quantify viral genetic material. Unless full-length complete genome amplification is demonstrated after a nuclease treatment, it should be emphasized that the detection of viral genomic material by qPCR or RT-PCR is not suitable to confirm the presence of infectious viral particles. This is because qPCR or RT-PCR can detect a fragment of the viral genome even in situations where no complete genomes and/or infectious viral material are present. To have a better insight into the potential for transmission, it is recommended to perform infectivity assays involving the in vitro culturing of shed material with a permissive cell line or growth tests (e.g., plaque assay) if qPCR results reach a level above the limit of detection (LOD) [25,26,27]. qPCR or RT-PCR results should be accompanied with the determination of the LOD and limit of quantification (LOQ) to enable an evaluation of the sensitivity and the reliability of the assay. Also, the inclusion of suitable controls (e.g., spiking with a reference standard or an internal positive control) should be considered to account for possible effects that could lead to an underestimation of the level of shedding, for example due to the nature of the matrix of the biological sample.

Our review of the literature shows that many studies limit the collection of shedding data by conducting quantitative real-time PCR (qRT-PCR) analyses. Of the 70 clinical trials mentioning shedding analysis, 4 did not mention the method. In 32 of the clinical trials, only a PCR test was performed, while 14 of the studies did only perform an infectivity test such as a cell culture or a plaque assay test. In 17 of the trials, both a PCR and a cell culture or plaque assay were performed.

In most of the clinical trials that performed infectivity tests alone or in combination with a PCR, no infectious particles were observed in the various shedding samples analyzed. However, in a few trials (6/32), the presence of infectious particles was shown by cell culture or plaque assay in some shedding samples. This was the case for some saliva samples from metastatic prostate cancer subjects treated with a high dose of the oncolytic Adenovirus CG7870 [28], and for three subjects with solid tumors repeatedly treated intratumorally with the oncolytic Adenovirus ONCOS-102 and presenting infectious viral particles in buccal samples and, for one subject, also in the urine [29]. The presence of shed infectious particles in samples was also observed with other OV such as the Herpesvirus Imlygic^®®^, for which swabs from the surface of injected lesions from subjects with unresectable recurrent melanoma tested positive for infectivity [30], as well as the Parvovirus H-1, which was injected in subjects with metastatic pancreatic ductal adenocarcinoma (PDAC) and for which infectious particles were detected in feces swabs of five of the seven subjects [31]. We also observed the naturally Picornavirus Seneca Valley Virus (SVV-001), showing infectious virus in nasal secretions, sputum, blood, urine, and stool in all dose cohorts during the first weeks after injection [32], and the Vaccinia Virus (GL-ONC1), for which skin rash swabs were found positive for a virus in 2 out of 19 subjects with locoregionally advanced head and neck carcinoma [33]. All these examples illustrate that even if the shedding of infectious viral particles seems to be a rare event, it cannot be excluded.

### 4.3. Aspects of the ERA Impacting Shedding

A proper ERA of OV addresses several aspects that may impact the release of the viral particles by the host. This includes among others an examination of the genetic stability, the conditions under which replication is occurring, replication competence and the route of administration of the OV. Characteristics from the wild-type virus from which the OV has been derived, such as the pathogenicity, the tropism, the host range, the natural route of transmission and the shedding pattern, may provide valuable information to perform the ERA of OV. Furthermore, each of these aspects may be altered by the overall design and the genetic modification proper to a given OV [24,34].

**Figure 4:**
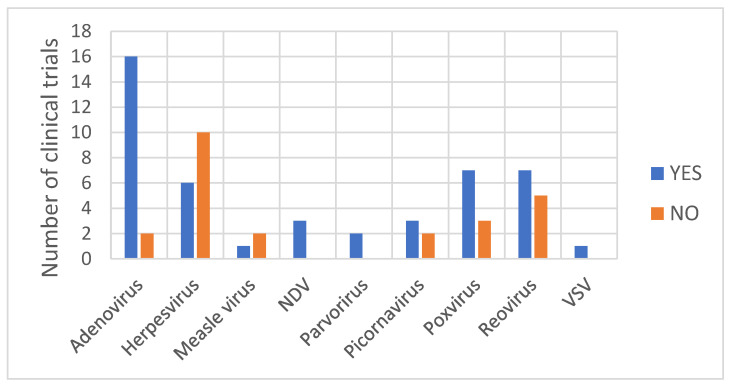
The number of clinical trials (by type of viral vector) for which the detection of viral genome was observed or was not observed in shedding samples.

With regard to the type of virus from which OV is derived, the detection of a viral DNA/RNA genome was observed at least once for all virus families (Figure 4). A rather clear picture is depicted for Adenovirus vectors, as the detection of the viral genome during shedding analysis was observed in 89% of the clinical trials with subjects treated with an adenoviral vector. It remains difficult to retrieve a general consideration for the other OV. As observed in Table 1, for oncolytic Herpesvirus vectors, the detection of the viral genome within shedding samples varies depending on the vector. Shedding has been observed only with the Herpesvirus Imlygic^®®^ [30] and OrienX010 [35]. No shedding has been observed with the Herpesvirus OH2 [36], G207 [37,38,39], G47Δ [40], HF10 [41] or HSV176 [42,43]. Imlygic^®®^ and OrienX010 both express the transgene GM-CSF used to boost the anti-tumor immune response by promoting dendritic cell recruitment and activation following tumor antigens’ liberation from lysing tumor cells. Although OH2 also expresses the transgene GM-CSF, no shedding of viral particles has been observed [36].

Differences in shedding pattern have also been observed within the Picornaviruses class. Shedding of the viral particles from subjects treated with the naturally occurring replication-competent Picornavirus, Coxsackievirus [47] or Seneca Valley virus [32,48] has been observed, whereas no shedding has been observed from subjects treated with the recombinant Poliovirus PVS-RIPO [49,50]. For other types of OV, an analysis of the shedding pattern does not reveal any general trends, partly due to the relatively low number of studies reporting shedding analysis results.

In addition to the intrinsic properties of the OV and irrespective of the type of virus, due account should be given to the interaction of the OV with the host, which may have an effect on this interaction and hence on the shedding pattern. One should therefore remain cautious with extrapolating pre-clinical data to human beings. For example, the results of quantitative real-time PCR (qRT-PCR) indicate that VSV-IFNβ-NIS RNA was detectable in some early nasal, oral, and rectal swabs of inoculated pigs [51], or in some buccal swabs, urine or fecal samples of inoculated cancer dogs with detectable VSV-N gene copies close to or below the limit of detection (LOD) [52], with no infectious virus detected in any collected samples. These data are consistent with shedding results obtained during a clinical trial with subjects with hematologic malignancies intravenously injected with VSV-IFNβ-NIS. Quantitative RT-PCR at day 2 revealed a low level of viral genome in the saliva with no infectious virus detected [53]. Although, in non-clinical studies in non-human primates, presenting many similarities with humans, qRT-PCR analysis revealed no detection of viral genome in shedding buccal swab samples from these animals treated with the oncolytic virus VSV-IFNβ [54].

**Figure 5:**
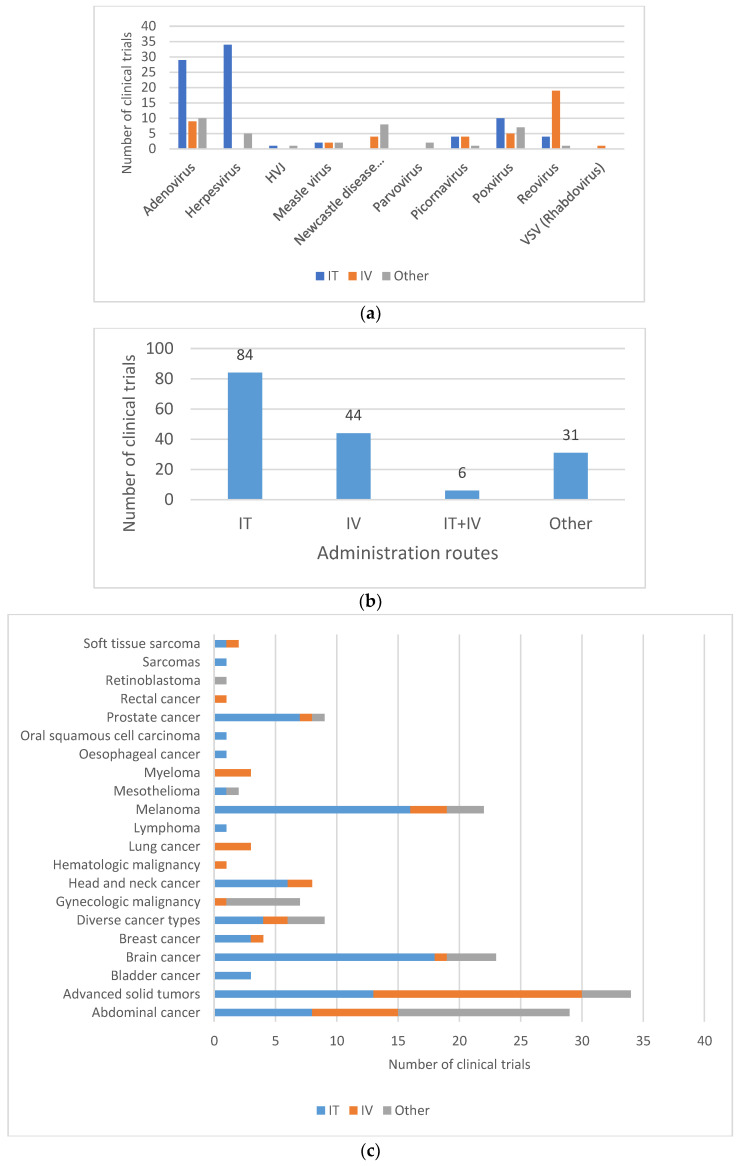
Routes of administration for oncolytic viruses in clinical trials. (**a**) Route of administration by type of oncolytic vectors. (**b**) Most commonly used administration routes in clinical trials. Most virus deliveries were performed by intratumoral route (*n* = 84). Other routes include, among others, intradermal injection (8/165), intramuscular injection (1/165) and hepatic arterial injection (4/165). (**c**) Route of administration by type of cancer. IT = intratumoral; IV = intravenous. Sarcomas also include soft-tissue sarcoma. Brain cancers include central nervous system (CNS) cancer, glioma and glioblastoma. Gynecologic malignancies include ovarian cancer, tubal cancer, endometrial cancer or peritoneal cancer. Abdominal cancers include liver cancer, colorectal cancer, pancreatic cancer, kidney cancer (renal cell cancer) and stomach cancer (gastric cancer).

The route of administration used during clinical application usually differs from the natural portal of entry of the wild-type virus from which the OV are derived, and may also impact shedding pattern (Figure 5A,B). Intraperitoneal, intratumoral, intravenous or hepatic arterial injections are all routes of administration that may change the biodistribution and the shedding properties of the OV. In a phase I study with subjects with advanced solid tumors, treated with the replicating Adenovirus ONCOS-102, quantifiable levels of viral genomes were found in urine and buccal swabs after treatment, among which three subjects were found positive for infective virus 3 days after the first intravenous administration with 20% of the dose. However, all samples were found negative when the entire dose of ONCOS-102 was given intratumorally [29]. In another study investigating the safety and tolerability of an oncolytic H-1 Parvovirus, subjects were assigned to two arms differing in the route of administration of the initial virus application consisting either of a single intratumoral injection or five intravenous virus infusions on days 1 to 5. While in the intratumorally injected subjects, the viral genome was only detected in fecal samples at the highest dose, feces samples of all but one of the intravenously injected subjects were found positive at the lower doses [55]. No shedding of Measles virus MeV-NIS was observed when administrated intraperitoneally in subjects with ovarian cancer [56], whereas shedding was observed when administrated intravenously to subjects with recurrent or refractory multiple myeloma [57].

The same observation can be made with two oncolytic Poxvirus GL-ONC1 and Pexa-Vec. Shedding was observed when GL-ONC1 was delivered intravenously to subjects with advanced head and neck carcinoma [33], whereas no shedding was observed with the intraperitoneal injection of GL-ONC1 [58]. In a phase I clinical trial with intratumoral injection of the modified poxvirus Pexa-Vec/JX-594 into subjects with refractory primary or metastatic liver cancer, no evidence of viral genome shedding was observed by plaque assay analyses of urine and throat swab samples [59]. However, in a phase IIb clinical trial with Pexa-Vec given as a single IV infusion followed by up to five IT injections in subjects with advanced hepatocellular carcinoma, Pexa-Vec was recovered from throat swabs at day 8 post-IV (and before IT injection) in 36% (9/25) of the subjects, but not thereafter. All urine samples were tested negative at all timepoints [60]. Hence, it is important to consider that the location of the cancer and the concomitant choice of delivery of the investigated OV may affect subsequent shedding.

For oncovirotherapy, direct intratumoral injection is a preferred route of administration for brain tumors (18/23), despite the fact that it may pose significant challenges. Intratumoral injection of the OV is also mainly used for easily injectable tumors, such as melanoma (16/22), or head and neck cancers (6/8) (Figure 5C).

OV are also often administered intravenously in subjects with metastatic tumors. In this case, OV encounter many physiological barriers before reaching cancer lesions and repeated doses may be necessary, thereby triggering the recognition and attack by the immune system and clearance of the OV by neutralizing antibodies. While the latter is a concern for efficacy reasons, elicitation of a strong immune response may also result in a shorter duration of shedding.

As has been observed by Dunn et al. [61] and Weil et al. [62], the immune status of the subject could also have an effect on viral replication and subsequent viral genome shedding. Therefore, another aspect that could influence the viral vector shedding pattern is the use of concomitant drugs or treatments (e.g., radiotherapy, chemotherapy). Given the heterogeneity of cancer types, OV are likely to be administrated as part of combination regimens involving the modulation of immuno-inhibitory pathways and the T lymphocyte activation. Roulstone et al. reported that the shedding of RT3D RNA was more frequent when Reovirus was administrated together with cyclophosphamide than with Reovirus alone, or with combination regimens of RT3D and conventional chemotherapy [63].

Finally, the reactivation of latent OV, influenced by the immune status of the subject, could possibly lead to a shedding pattern that is different from what could have been predicted from former clinical experiences. For oncolytic viruses with the potential for latency reactivation, the collection of additional samples for shedding analysis when subjects show signs of infection due to reactivation could be planned.

### 4.4. Shedding Study Design

Each of the above-mentioned elements contributes to a risk-based approach in the design and the extent of shedding studies. It may provide insights into the choice of biological samples to be analyzed, as well as the frequency and the duration of monitoring.

**Figure 6:**
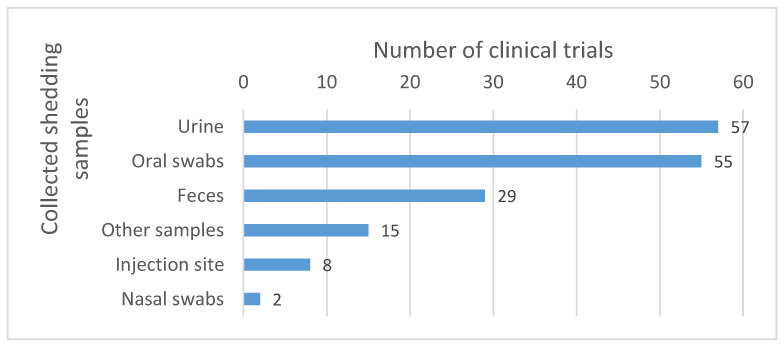
Body fluids collected for the shedding analysis. The numbers reported in the graph correspond to the number of clinical trials in which the corresponding samples were collected. Other samples include, among others, dressing swabs, lesion swabs, rash swabs, pustules swabs, etc.

Samples most commonly collected include urine and oral swabs. Other sample types such as feces, nasal swabs and injection site samples are also collected, but less commonly (see Figure 6).

The collection of data should also take into account the occurrence of metastasis. For example, secondary tumors may also be located in the oral cavity, larynx, pharynx or esophagus, justifying the collection of saliva samples.

When OV are administrated by the intradermal route, there is a possibility of transmission of the OV through skin contact. Therefore, the collection of skin swabs at the site of injection, in addition to samples routinely assessed for shedding, should be recommended in order to determine whether an appropriate occlusive dressing is required as a precautionary measure. According to our analysis, none of the trials performing intradermal injection reported shedding results. On the other hand, eight trials where the OV were administrated intratumorally included swabs from the injection site in their shedding analysis. From these eight trials, shed viral DNA at the injection site was observed in five trials. Of these five clinical trials, four trials were performed with Imlygic^®®^ [30,64,65,66] and one with the Adenovirus Ad.hIFN-beta [67]. Detectable Imlygic^®®^ DNA was observed on the surface of injected lesions for all treated subjects, and it was still detected for 14% of subjects during the safety follow-up period. Of the 740 swabs from the surface of injected lesions, 8 tested positive for infectivity [30]. The Adenovirus Ad.hIFN-beta DNA was detected and remained in the injection site area for at least 8 days [67].

The duration and frequency of sample collection should be decided on a case-by-case basis, taking into account the characteristics of the parental virus, the conditionally replicative competence of the OV and the immune status of the subject. As most of the OV are still replication-competent, the duration of sample collection should also take into account the possible appearance of a secondary peak of shedding. As one of the numerous examples of viral replication, it was shown that in subjects treated with the intratumorally injected HSP70, a telomerase-specific replication-competent oncolytic Adenovirus (telomelysin), viral DNA was detectable in plasma or sputum at days 7 and 14 post-treatment, despite being below detectable levels at 24 h post-treatment [68].

### 4.5. Risk Management Measures

The identification and characterization of potential adverse effects associated with the use of a given OV and the assessment of their likelihood of occurrence may lead to the identification of potential risks. It is also possible that the case-specific assessment reveals remaining uncertainties, precluding any conclusion on the risks for human health and the environment. In the first case, the set-up and implementation of risk management measures aim at minimizing identified risks, while in the second case, these could serve as precautionary measures.

In cases wherein data support the low likelihood of shedding or where no risk related to the shedding is identified for human health or the environment, good working practices involving proper hand hygiene, personal protective equipment (PPE) and proper decontamination and waste procedures at the clinical setting will be effective to limit the inadvertent exposure of personnel and the possible dissemination of the OV into the environment (Table 2). The correct implementation of these measures necessitates personnel trained and experience in handling infectious material.

**Figure 7:**
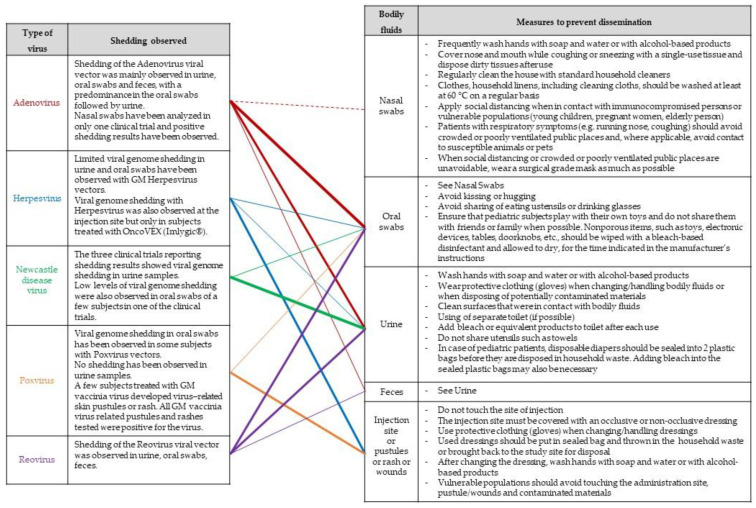
Possible risk-mitigation measures. The lines indicate observed trends in shedding results obtained from clinical trials involving OV. The thickness of the lines is proportional to shedding results per OV type.

When shedding by the treated subject cannot be excluded and potential risks for human health and the environment have been identified, risk management measures should also focus on minimizing the exposure of third parties outside the clinical setting, thereby giving particular attention to immunosuppressed or any vulnerable people (e.g., pregnant women, newborns, infants, elderly people). A list of possible measures that may be considered, taking into account the considerations made as part of a case-specific risk assessment, is provided in a guidance document endorsed by several national competent authorities involved in the risk assessment GMOs [27] and has been further adapted in Figure 7.

Geletneky et al. indicated in their article that glioblastoma subjects treated with the oncolytic Parvovirus H-1 were obliged to stay isolated in the study center until shed viral genomes were no longer detected in feces, urine or saliva, or until the first occurrence of H-1-specific antibodies [55].

It could be hypothesized that OV applications that have entered the market and that have successfully gone through the several stages of clinical development would rely on shedding data to determine risk management measures, if deemed relevant according to a proper risk assessment, and that data on the occurrence of transmission to close contacts would allow further refinement of risk management measures. In this ideal scenario, evidence-based data would contribute to a set of management measures that are as proportionate as possible to the environmental risk. However, the occurrence of secondary infections is barely addressed during clinical development. Given that these viral vectors are often derived from infectious viruses, any studies that contribute to the assessment of possible secondary infection would further contribute to the ERA of the medicinal product.

During a phase II clinical trial with modified Herpesvirus Imlygic^®®^, Andtbacka et al. reported that some mucosal or skin lesions were observed in close contact, and investigators all tested negative for T-VEC. However, none of the lesions were tested for wild-type HSV-1 or HSV-2, leaving an uncertainty as to the origin of the lesion [30].

The precautionary measures that were implemented for the subject during this phase II study with Imlygic^®®^ are also found in the current subject brochure for Imlygic^®®^, which stipulates measures to be taken by the subject to avoid the direct contact of thirds with the subjects’ bodily fluids or injection sites, such as covering injection sites with airtight and watertight dressings, implementing a proper disposal of used dressings and cleaning material to prevent household contacts from directly touching them [69].

An ongoing post-marketing study of melanoma subjects treated with Imlygic^®®^, started in 2017, is among others counting the numbers of herpetic infections, with the detection of T-DNA among close contacts and healthcare providers as a secondary outcome [70]. This exemplifies that efforts to collect information on the effective transmission of Imlygic^®®^ are being pursued.

Finally, if animals may be infected, appropriate measures to limit exposure to susceptible pets or other animals in the immediate surroundings of the treated subject should be considered. Newcastle disease virus (NDV), for example, is a naturally occurring oncolytic virus that causes severe illness in birds and poultry, and could therefore pose an environmental risk even if it is non-pathogenic in humans. In three clinical trials with intravenously injected NDV-PV701 in subjects with advanced solid cancer [71,72,73], low levels of viral genome shedding in urine have been observed up to 3 weeks after injection. Pecora et al. [73] also observed low and transient levels of viral genome shedding in sputum. Although, Pecora et al. suggested that the levels of shed PV701 are orders of magnitude below the standard avian vaccine dose required for an antibody response, appropriate biosafety measures to prevent environmental spread of the virus should be considered when administrating high-dose oncolytic NDV. Subjects working with birds and poultry or subjects having a pet bird or poultry at home could be asked to avoid contact with these NDV host species for a certain time after injection of the IMP, in order to reduce the potential environmental impact of viral shedding on the most susceptible host species.

Another example of clinical trials where measures to avoid contact with animals have been proposed involved the use of a recombinant chimeric Vesicular stomatitis virus, in which the VSV glycoprotein was replaced to abrogate neurotoxicity and pathogenicity [74]. As the OV were derived from a vector-borne virus causing significant disease in pigs, cattle and horses, instructions for avoiding contact with livestock for seven days following administration of the OV have been proposed [75].

## 5. Discussion and Recommendations

The inventory presented in this paper provides a state-of-the-art of the shedding analysis of OV in clinical practice, and raises the question: to what extent it is possible to build upon the experience gained so far with shedding data to draw conclusions for each of the different types of OV? This may be particularly relevant in cases where historical shedding data have been obtained for well-characterized OV, of which the designs present relevant similarities with a novel investigational OV. Our literature search reveals that in 89% of the studies with Adenovirus-derived OV reporting shedding analysis, positive shedding results were obtained. However, as illustrated above by the several examples developed in this paper, the shedding patterns remain diverse and complex, as well as for Adenovirus-derived OV, thereby hindering the development of a standardized study design. Diversity in shedding patterns was observed even when using OV derived from the same type of virus. A number of elements may impact the shedding pattern, such as the specific design of the viral vector and the transgene harbored by the viral vector, the interaction of the OV with their host organisms, the immune status of the patient or the variety of the clinical protocols, such as differences in the administration route or the use of concomitant drugs.

Valuable information and insights into the toxicity, biodistribution and shedding pattern of OV could be obtained from non-clinical studies. As compared to clinical studies, animal studies are more amenable to be conducted in the early stages of the development of investigational OV. However, it should be taken into account that data are not readily extrapolable from animals to human, in particular when different routes of administration are used or no data have been collected in larger animals, such as non-human primates. Also, an animal model fails to mimic the patient-specific immune status. This means that an absence of viral shedding in animal studies does not necessarily preclude viral shedding in humans, as a different host organism may induce a different behavior of the virus (viral replication, viral clearance, viral tropism).

Because the shedding pattern strongly depends on different factors and because it can also differ between animals and humans, the collection of shedding data in the earliest phase of clinical development of investigational OV is strongly recommended regardless of the OV vector.

The FDA guidance for the design and analysis of shedding studies also recommends the collection of shedding data on OV in the earliest phase of clinical development (phase I) and anytime afterwards if the dose, the route of administration, the regimen or the indication are modified [25]. Likewise, both an EMA guideline on scientific requirements for the ERA of Gene Therapy Medicinal Products and an EC consideration document specifically addressing the evaluation of shedding with OV recommend addressing shedding analysis as early as possible in the clinical development, and more particularly during a phase I study [23,26]. Whilst the time point of sample collection for shedding has not been further specified in the EC consideration document, the FDA guidance recommends sampling on days 1, 3, 7 and 10, and then weekly, until the shedding analysis reveals three consecutive results below the LOD of the assay. Notably, all guidance emphasizes the need of a case-by-case approach taking into account the properties of the OV (replication competence, known persistence or possibility of latency reactivation of the parental virus from which the OV are derived) and the interaction with the host (immune status of the patient and thirds, single versus multiple round of administration and effect on clearance by the immune system) [21,23,25,26]. For example, in the case of subsequent rounds of dose administration, the time point of sampling can be adapted when justified by a proper shedding analysis obtained with a single-dose administration.

A possible concern, for which we could not find experimental data, is the likelihood of an in vivo recombination between the OV and endogenous viruses circulating in the trial participants. This assessment should not be neglected because recombination events could lead to the formation of uncharacterized variants that could be more virulent and that could affect the shedding pattern and the potential for transmission. These newly generated viruses could therefore compromise the environmental safety. In general, the likelihood of the recombination between viruses significantly increases with the prevalence of co-circulating viruses in the population, and with their genetic homology. High viral loads, often a relevant feature of replication-competent OV, increase the chance of exchanging strands, explaining why, in many cases, recombination is often replication-dependent [76]. Moreover, Buijs et al. [77] mentioned the relevance of assessing the recombination of these OV with wild-type viruses given the ongoing strategies to use more virulent conditionally replicating viruses. A possible explanation for the fact that the likelihood of the recombination of OV has been barely examined is that, unless there is scientific evidence pointing to recombination, such as in in vitro experiments demonstrating the generation of novel and uncharacterized recombinants, developers could be hesitant to pursue this research due to the anticipated low occurrence and the technical hurdles of the lower limit of detection and quantification associated with the monitoring of viral particles.

Whilst the collection of shedding data undoubtedly supports an evidence-based ERA, it is important to be aware that the shedding of OV or any other viral vector or virus does not necessarily involve a risk. Potential adverse effects for close contacts or the environment that could arise following shedding do not only result from the presence of viral particles in the shedding samples, but also depend on the stability of the shed viral particles under environmental conditions outside the host, the route of transmission (e.g., spreading through aerosols, fecal–oral route of transmission via direct contact or contaminated fluids, vector-borne transmission, through parenteral exposure), the capacity of the shed particles to infect cells of other persons or animals, and, as a last element in the chain of events for environmental risk to occur, the capacity of the OV to cause adverse effects in the novel host organism. To possibly alleviate remaining uncertainties in secondary transmission, it is therefore important to answer the questions of whether the observed shed particles are only vector DNA/RNA or a remnant thereof, and whether these shed particles are still infectious. These observations will contribute to a proportionate risk management by allowing the determination of appropriate precautionary measures.

Indeed, a fundamental question when uncertainties remain regarding the actual risk associated to the shedding of OV is what level of risk management measures should be taken, or what level of uncertainty would warrant precautionary measures. If it remains unclear whether shedding may occur and what risks it may entail for human health and the environment, a drastic and conservative scenario would be to eliminate any possibility of release of OV into the environment by keeping patients for several weeks/months in the hospital setting. However, the implementation of stringent measures may carry drawbacks, as it may increase costs and time not only for the appropriate training of personnel, the set-up and follow-up of waste disposal procedures and logistics, but also for the recruitment of subjects requiring their informed consent and the training of close contacts if the trial participant is discharged. Ideally, risk management measures should be as proportionate as possible to the actual risk posed so as not to hinder the development of research and innovation and to safeguard patient access to innovative treatments, while ensuring the proper protection of human health at large and the environment. A way to contribute to proportionate risk management is to continue to gathering data-based evidence by including within the shedding analysis the determination of the fraction of infectious particles in early phases of the clinical development of OV.

## 6. Concluding Recommendations

With the diversity of OV that entered the clinical research and development landscape, this work demonstrates the current gaps in data-based evidence on shedding and the challenge of defining risk management measures that are proportionate to the actual risk posed for human health and the environment. In accordance with GMO legislation requiring a case-specific and risk-proportionate approach, this paper aims at encouraging the collection of shedding data as early as possible in the developmental plan in the rapidly growing area of OV. The demonstration of the shedding of infectious particles may warrant assessments of the potential of secondary transmission. The collection of real-world transmission data is expected to provide a better understanding of transmissibility, which is key to characterizing the risk for the human population and the environment. It will also benefit future clinical trials developers in establishing a clinical protocol based on evidence-based risk assessment.

## Figures and Tables

**Table 1:** Overview of different oncolytic Herpesvirus vectors for which shedding analyses have been performed and the shedding observation.

Herpesvirus Vectors	Genetic Modifications [44]	Administration Route	Viral Genome Shedding	References
Imlygic^®®^	Armed recombinant HSV ^1^ with GM-CSF ^2^ transgen	IT ^3^	Yes	[30]
OrienX010	Armed recombinant HSV with GM-CSF transgen	IT	Yes	[35]
OH2	Armed recombinant HSV with GM-CSF transgen	IT	No	[36]
G207	Conditionally replicating HSV with multiple mutations	IT	No	[37,38,39]
G47Δ	Conditionally replicating HSV with multiple mutations	IT	No	[40]
HF10	Conditionally replicating HSV with multiple mutations	IT	No	[41]
HSV1716	Conditionally replicating HSV with multiple mutations	IT	No	[42,43]
NV1020	Conditionally replicating HSV with multiple mutations	Hepatic arterial injection	Yes and No	[45,46]

^1^ HSV: Herpes Simplex Virus; ^2^ GM-CSF: Granulocyte-macrophage colony-stimulating factor; ^3^ IT: intratumoral injection.

**Table 2:** Examples of good working practices for personnel manipulating oncolytic vectors to prevent or manage risks for human health and/or the environment.

Preventing Measures	PPE	- Always use a lab coat and gloves to avoid any skin contamination during OV preparation and administration - Workers should wear a mask that conforms with the norm NBN EN 529, a FFP2 type (EN149:2001) with a P2 filter (EN 143:2000)
	Needle preparation	- The needle preparation of vials containing the oncolytic vector may generate aerosols. The preparation of the OV for administration is recommended to be conducted in a class II Biosafety Cabinet. Otherwise, the use of goggles and masks should be mandatory during the puncture of the vial and needle preparation - Removal of the syringe should occur by means of hands-free operation (i.e., hands do not touch the needle) into a closed container
	Spill kits	- Spill kits containing materials for spill clean-up should be on hand (or must be easily accessible to personnel) before handling the OV - The spill kit should contain liquid disinfectant, personal protective equipment (i.e., gloves, safety glasses, laboratory coat, shoe covers, mask), absorptive paper towels, tongs or forceps, a sharp container and biohazard waste bags
Risk Management Measures	Skin contact	- Skin contamination through spills should be handled by first placing an absorbent tissue on the affected area to adsorb all viral particles. An effective disinfectant should then be applied to the tissue. After removing the tissue, the skin should be washed with soap and water thoroughly and the tissue should be disposed of as a biohazard material
	Mucus membrane or eye contact	- In the case of accidental contact with open mouth or eye, rinse mouth or eye thoroughly over a closing basin. The collected washing liquid should be decontaminated with appropriate disinfectant before disposal - In the case of accidental ingestion, do not induce vomiting
	Accidental spill	In the case of accidental spills or breakage of a vial containing the GMO: - People in the area of the spill should be alerted and asked to leave the area - All personnel involved with the spill should remove contaminated clothes before leaving the area - The area should be closed to allow aerosols to be carried away and heavier particles to settle and a message “DO NOT ENTER” should be posted - After 30 min, the area can be entered again by wearing a clean lab coat, disposable gloves, glasses, disposable shoe covers and a mask - The spill should be covered with towels or other absorbent material starting from the edge toward the center. Appropriate disinfectant should be poured over the absorbent material starting from the edge to the center. Sufficient contact time should be allowed so as to ensure inactivation of the GMO by the disinfectant - After that, the paper towels and broken vials should be removed with tongs or forceps and discarded in a biohazard waste bag. The PPE should be discarded in the biohazard bag. The lab coat should be decontaminated before disposal - The medical staff should report the incident to the responsible member on the site

## Data Availability

Data sharing not applicable.

## Supplementary Materials

The following supporting information can be downloaded at: https://www.mdpi.com/article/10.3390/vaccines11091448/s1, Table S1: Final list of 165 manuscripts.
