# Supplementary material for: Oncolytic Viruses: An Inventory of Shedding Data from Clinical Trials and Elements for the Environmental Risk Assessment
Source: Vaccines (Basel). 2023 Sep 1;11(9):1448. doi: 10.3390/vaccines11091448 (PMC10535390; doi:10.3390/vaccines11091448)
Supplement: Supplementary file 1 [file vaccines-11-01448-s001.zip › vaccines-2530227-supplementary.pdf]

Table S1. Final list of 165 manuscripts

PMID	Study
30179611	Alberts P, Tilgase A, Rasa A, Bandere K, Venskus D. The advent of oncolytic virotherapy in oncology: The Rigvir® story. Eur J Pharmacol. 2018 Oct 15;837:117–26.
31409575	Andtbacka RHI, Amatruda T, Nemunaitis J, Zager JS, Walker J, Chesney JA, et al. Biodistribution, shedding, and transmissibility of the oncolytic virus talimogene laherparepvec in patients with melanoma. EBioMedicine. 2019 Sep;47:89–97.
31171039	Andtbacka RHI, Collichio F, Harrington KJ, Middleton MR, Downey G, Öhrling K, Kaufman HL. Final Analyses of OPTiM: A Randomized Phase III Trial of Talimogene Laherparepvec versus Granulocyte-Macrophage Colony-Stimulating Factor in Unresectable Stage III-IV Melanoma. J Immunother Cancer. 2019Jun6;(7):145.
31273010	Annels NE, Mansfield D, Arif M, Ballesteros-Merino C, Simpson GR, Denyer M, et al. Phase I Trial of an ICAM-1-Targeted Immunotherapeutic-Coxsackievirus A21 (CVA21) as an Oncolytic Agent Against Non Muscle-Invasive Bladder Cancer. Clin Cancer Res. 2019 Oct 1;25(19):5818–31.
35149591	Bazan-Peregrino M, Garcia-Carbonero R, Laquente B, Álvarez R, Mato-Berciano A, Gimenez-Alejandre M, et al. VCN-01 disrupts pancreatic cancer stroma and exerts antitumor effects. J Immunother Cancer. 2021 Nov;9(11):e003254.
33875611	Beasley GM, Nair SK, Farrow NE, Landa K, Selim MA, Wiggs CA, et al. Phase I trial of intratumoral PVSRIPO in patients with unresectable, treatment-refractory melanoma. J Immunother Cancer. 2021 Apr;9(4):e002203.
29027598	Bernstein V, Ellard SL, Dent SF, Tu D, Mates M, Dhesy-Thind SK, et al. A randomized phase II study of weekly paclitaxel with or without pelareorep in patients with metastatic breast cancer: final analysis of Canadian Cancer Trials Group IND.213. Breast Cancer Res Treat. 2018 Jan;167(2):485–93.
2208003	Bohle W, Schlag P, Liebrich W, Hohenberger P, Manasterski M, Möller P, et al. Postoperative active specific immunization in colorectal cancer patients with virus-modified autologous tumor-cell vaccine. First clinical results with tumor-cell vaccines modified with live but avirulent Newcastle disease virus. Cancer. 1990 Oct 1;66(7):1517–23.
29748010	Bradbury PA, Morris DG, Nicholas G, Tu D, Tehfe M, Goffin JR, et al. Canadian Cancer Trials Group (CCTG) IND211: A randomized trial of pelareorep (Reolysin) in patients with previously treated advanced or metastatic non-small cell lung cancer receiving standard salvage therapy. Lung Cancer. 2018 Jun;120:142–8.
21886163	Breitbach CJ, Burke J, Jonker D, Stephenson J, Haas AR, Chow LQM, et al. Intravenous delivery of a multi-mechanistic cancer-targeted oncolytic poxvirus in humans. Nature. 2011 Aug 31;477(7362):99–102.
26072416	Breitbach CJ, Moon A, Burke J, Hwang TH, Kirn DH. A Phase 2, Open-Label, Randomized Study of Pexa-Vec (JX-594) Administered by Intratumoral Injection in Patients with Unresectable Primary Hepatocellular Carcinoma. Methods Mol Biol. 2015;1317:343–57.

23088985	Burke JM, Lamm DL, Meng MV, Nemunaitis JJ, Stephenson JJ, Arseneau JC, et al. A first in human phase 1 study of CG0070, a GM-CSF expressing oncolytic adenovirus, for the treatment of nonmuscle invasive bladder cancer. J Urol. 2012 Dec;188(6):2391–7.
25307519	Burke MJ, Ahern C, Weigel BJ, Poirier JT, Rudin CM, Chen Y, et al. Phase I trial of Seneca Valley Virus (NTX-010) in children with relapsed/refractory solid tumors: a report of the Children's Oncology Group. Pediatr Blood Cancer. 2015 May;62(5):743–50.
21673660	Cerullo V, Diaconu I, Kangasniemi L, Rajecki M, Escutenaire S, Koski A, et al. Immunological effects of low-dose cyclophosphamide in cancer patients treated with oncolytic adenovirus. Mol Ther. 2011 Sep;19(9):1737–46.
19242097	Chang J, Zhao X, Wu X, Guo Y, Guo H, Cao J, et al. A Phase I study of KH901, a conditionally replicating granulocyte-macrophage colony-stimulating factor: armed oncolytic adenovirus for the treatment of head and neck cancers. Cancer Biol Ther. 2009 Apr;8(8):676–82.
29176501	Chesney J, Awasthi S, Curti B, Hutchins L, Linette G, Triozzi P, et al. Phase IIIb safety results from an expanded-access protocol of talimogene laherparepvec for patients with unresected, stage IIIB-IVM1c melanoma. Melanoma Res. 2018 Feb;28(1):44–51.
28981385	Chesney J, Puzanov I, Collichio F, Singh P, Milhem MM, Glaspy J, et al. Randomized, Open-Label Phase II Study Evaluating the Efficacy and Safety of Talimogene Laherparepvec in Combination With Ipilimumab Versus Ipilimumab Alone in Patients With Advanced, Unresectable Melanoma. J Clin Oncol. 2018 Jun 10;36(17):1658–67.
35998300	Chesney JA, Ribas A, Long GV, Kirkwood JM, Dummer R, Puzanov I, Hoeller C, Gajewski TF, Gutzmer R, Rutkowski P, et al. Randomized, Double-Blind, Placebo-Controlled, Global Phase III Trial of Talimogene Laherparepvec Combined With Pembrolizumab for Advanced Melanoma. J Clin Oncol. 2023 Jan20;41(3):528–540.
36875061	Chintala NK, Choe JK, McGee E, Bellis R, Saini JK, Banerjee S, Moreira AL, Zauderer G, Adusumilli PS, Rusch VW. Correlative Analysis from a Phase I Clinical Trial of Intrapleural Administration of Oncolytic Vaccinia Virus (Olvi-Vec) in Patients with Malignant Pleural Mesothelioma. Front Immunol. 2023 Feb ;14 :1112960.
15509513	Chiocca EA, Abbed KM, Tatter S, Louis DN, Hochberg FH, Barker F, et al. A phase I open-label, dose-escalation, multi-institutional trial of injection with an E1B-Attenuated adenovirus, ONYX-015, into the peritumoral region of recurrent malignant gliomas, in the adjuvant setting. Mol Ther. 2004 Nov;10(5):958–66.
18180770	Chiocca EA, Smith KM, McKinney B, Palmer CA, Rosenfeld S, Lillehei K, et al. A phase I trial of Ad.hIFN-beta gene therapy for glioma. Mol Ther. 2008 Mar;16(3):618–26.
28756871	Cohn DE, Sill MW, Walker JL, O'Malley D, Nagel CI, Rutledge TL, et al. Randomized phase IIB evaluation of weekly paclitaxel versus weekly paclitaxel with oncolytic reovirus (Reolysin®) in recurrent ovarian, tubal, or peritoneal cancer: An NRG Oncology/Gynecologic Oncology Group study. Gynecol Oncol. 2017 Sep;146(3):477–83.

20926400	Comins C, Spicer J, Protheroe A, Roulstone V, Twigger K, White CM, et al. REO-10: a phase I study of intravenous reovirus and docetaxel in patients with advanced cancer. Clin Cancer Res. 2010 Nov 15;16(22):5564–72.
35175355	Cook J, Peng KW, Witzig TE, Broski SM, Villasboas JC, Paludo J, et al. Clinical activity of single-dose systemic oncolytic VSV virotherapy in patients with relapsed refractory T-cell lymphoma. Blood Adv. 2022 Jun 14;6(11):3268–79.
25531693	Cripe TP, Ngo MC, Geller JI, Louis CU, Currier MA, Racadio JM, et al. Phase 1 study of intratumoral Pexa-Vec (JX-594), an oncolytic and immunotherapeutic vaccinia virus, in pediatric cancer patients. Mol Ther. 2015 Mar;23(3):602–8.
35383116	Cui C, Wang X, Lian B, Ji Q, Zhou L, Chi Z, et al. OrienX010, an oncolytic virus, in patients with unresectable stage IIIC-IV melanoma: a phase Ib study. J Immunother Cancer. 2022 Apr;10(4):e004307.
36564126	Curti BD, Richards J, Hyngstrom JR, Daniels GA, Faries M, Feun L, Margolin KA, Hallmeyer S, Grose M, Zhang Y, et al. Intratumoral Oncolytic Virus V937 plus Ipilimumab in Patients with Advanced Melanoma: The Phase 1b MITCI Study. J Immunother Cancer. 2022 Jun;10(12) :e005224.
33160198	Danson SJ, Conner J, Edwards JG, Blyth KG, Fisher PM, Muthana M, et al. Oncolytic herpesvirus therapy for mesothelioma - A phase I/IIa trial of intrapleural administration of HSV1716. Lung Cancer. 2020 Dec;150:145–51.
16829761	Dempsey MF, Wyper D, Owens J, Pimlott S, Papanastassiou V, Patterson J, et al. Assessment of 123I-FIAU imaging of herpes simplex viral gene expression in the treatment of glioma. Nucl Med Commun. 2006 Aug;27(8):611–7.
29943666	Desjardins A, Gromeier M, Herndon JE, Beaubier N, Bolognesi DP, Friedman AH, et al. Recurrent Glioblastoma Treated with Recombinant Poliovirus. N Engl J Med. 2018 Jul;12;379(2):150–61.
11606381	DeWeese TL, van der Poel H, Li S, Mikhak B, Drew R, Goemann M, et al. A phase I trial of CV706, a replication-competent, PSA selective oncolytic adenovirus, for the treatment of locally recurrent prostate cancer following radiation therapy. Cancer Res. 2001 Oct 15;61(20):7464–72.
28439108	Dispenzieri A, Tong C, LaPlant B, Lacy MQ, Laumann K, Dingli D, et al. Phase I trial of systemic administration of Edmonston strain of measles virus genetically engineered to express the sodium iodide symporter in patients with recurrent or refractory multiple myeloma. Leukemia. 2017 Dec;31(12):2791–8.
27203445	Downs-Canner S, Guo ZS, Ravindranathan R, Breitbach CJ, O'Malley ME, Jones HL, et al. Phase 1 Study of Intravenous Oncolytic Poxvirus (vvDD) in Patients With Advanced Solid Cancers. Mol Ther. 2016 Aug;24(8):1492–501.
34608333	Dummer R, Gyorki DE, Hyngstrom J, Berger AC, Conry R, Demidov L, et al. Neoadjuvant talimogene laherparepvec plus surgery versus surgery alone for resectable stage IIIB-IVM1a melanoma: a randomized, open-label, phase 2 trial. Nat Med. 2021 Oct;27(10):1789–96.
25469725	El-Sherbiny YM, Holmes TD, Wetherill LF, Black EVI, Wilson EB, Phillips SL, et al. Controlled infection with a therapeutic virus defines the activation kinetics of human natural killer cells in vivo. Clin Exp Immunol. 2015 Apr;180(1):98–107.
34214495	Fares J, Ahmed AU, Ulasov IV, Sonabend AM, Miska J, Lee-Chang C, et al. Neural stem cell delivery of an oncolytic adenovirus in newly diagnosed

	malignant glioma: a first-in-human, phase 1, dose-escalation trial. Lancet Oncol. 2021 Aug;22(8):1103–14.
19018254	Fong Y, Kim T, Bhargava A, Schwartz L, Brown K, Brody L, et al. A herpes oncolytic virus can be delivered via the vasculature to produce biologic changes in human colorectal cancer. Mol Ther. 2009 Feb;17(2):389–94.
18253152	Forsyth P, Roldán G, George D, Wallace C, Palmer CA, Morris D, et al. A phase I trial of intratumoral administration of reovirus in patients with histologically confirmed recurrent malignant gliomas. Mol Ther. 2008 Mar;16(3):627–32.
16257582	Freeman AI, Zakay-Rones Z, Gomori JM, Linetsky E, Rasooly L, Greenbaum E, et al. Phase I/II trial of intravenous NDV-HUJ oncolytic virus in recurrent glioblastoma multiforme. Mol Ther. 2006 Jan;13(1):221–8.
12208748	Freytag SO, Khil M, Stricker H, Peabody J, Menon M, DePeralta-Venturina M, et al. Phase I study of replication-competent adenovirus-mediated double suicide gene therapy for the treatment of locally recurrent prostate cancer. Cancer Res. 2002 Sep 1;62(17):4968–76.
17375076	Freytag SO, Movsas B, Aref I, Stricker H, Peabody J, Pegg J, et al. Phase I trial of replication-competent adenovirus-mediated suicide gene therapy combined with IMRT for prostate cancer. Mol Ther. 2007 May;15(5):1016–23.
24837889	Freytag SO, Stricker H, Lu M, Elshaikh M, Aref I, Pradhan D, et al. Prospective randomized phase 2 trial of intensity modulated radiation therapy with or without oncolytic adenovirus-mediated cytotoxic gene therapy in intermediate-risk prostate cancer. Int J Radiat Oncol Biol Phys. 2014 Jun 1;89(2):268–76.
14612551	Freytag SO, Stricker H, Pegg J, Paielli D, Pradhan DG, Peabody J, et al. Phase I study of replication-competent adenovirus-mediated double-suicide gene therapy in combination with conventional-dose three-dimensional conformal radiation therapy for the treatment of newly diagnosed, intermediate- to high-risk prostate cancer. Cancer Res. 2003 Nov 1;63(21):7497–506.
33838625	Friedman GK, Johnston JM, Bag AK, Bernstock JD, Li R, Aban I, et al. Oncolytic HSV-1 G207 Immunovirotherapy for Pediatric High-Grade Gliomas. N Engl J Med. 2021 Apr 29;384(17):1613–22.
32112659	Fujita K, Kato T, Hatano K, Kawashima A, Ujike T, Uemura M, et al. Intratumoral and s.c. injection of inactivated hemagglutinating virus of Japan envelope (GEN0101) in metastatic castration-resistant prostate cancer. Cancer Sci. 2020 May;111(5):1692–8.
15647767	Galanis E, Okuno SH, Nascimento AG, Lewis BD, Lee RA, Oliveira AM, et al. Phase I-II trial of ONYX-015 in combination with MAP chemotherapy in patients with advanced sarcomas. Gene Ther. 2005 Mar;12(5):437–45.
25398436	Galanis E, Atherton PJ, Maurer MJ, Knutson KL, Dowdy SC, Cliby WA, et al. Oncolytic measles virus expressing the sodium iodide symporter to treat drug-resistant ovarian cancer. Cancer Res. 2015 Jan 1;75(1):22–30.
20103634	Galanis E, Hartmann LC, Cliby WA, Long HJ, Peethambaram PP, Barrette BA, et al. Phase I trial of intraperitoneal administration of an oncolytic measles virus strain engineered to express carcinoembryonic antigen for recurrent ovarian cancer. Cancer Res. 2010 Feb 1;70(3):875–82.
22871663	Galanis E, Markovic SN, Suman VJ, Nuovo GJ, Vile RG, Kottke TJ, et al. Phase II trial of intravenous administration of Reolysin(®) (Reovirus Serotype-3-dearing

	Strain) in patients with metastatic melanoma. Mol Ther. 2012 Oct;20(10):1998–2003.
35767439	Gállego Pérez-Larraya J, Garcia-Moure M, Labiano S, Patiño-García A, Dobbs J, Gonzalez-Huarriz M, et al. Oncolytic DNX-2401 Virus for Pediatric Diffuse Intrinsic Pontine Glioma. N Engl J Med. 2022 Jun 30;386(26):2471–81.
10741699	Ganly I, Kirn D, Eckhardt G, Rodriguez GI, Soutar DS, Otto R, et al. A phase I study of Onyx-015, an E1B attenuated adenovirus, administered intratumorally to patients with recurrent head and neck cancer. Clin Cancer Res. 2000 Mar;6(3):798–806.
30234393	García M, Moreno R, Gil-Martin M, Cascallò M, de Olza MO, Cuadra C, et al. A Phase 1 Trial of Oncolytic Adenovirus ICOVIR-5 Administered Intravenously to Cutaneous and Uveal Melanoma Patients. Hum Gene Ther. 2019 Mar;30(3):352–64.
35338084	Garcia-Carbonero R, Bazan-Peregrino M, Gil-Martín M, Álvarez R, Macarulla T, Riesco-Martinez MC, et al. Phase I, multicenter, open-label study of intravenous VCN-01 oncolytic adenovirus with or without nab-paclitaxel plus gemcitabine in patients with advanced solid tumors. J Immunother Cancer. 2022 Mar;10(3):e003255.
28923104	Garcia-Carbonero R, Salazar R, Duran I, Osman-Garcia I, Paz-Ares L, Bozada JM, et al. Phase 1 study of intravenous administration of the chimeric adenovirus enadenotucirev in patients undergoing primary tumor resection. J Immunother Cancer. 2017 Sep 19;5(1):71.
20486770	Geevarghese SK, Geller DA, de Haan HA, Hörer M, Knoll AE, Mescheder A, et al. Phase I/II study of oncolytic herpes simplex virus NV1020 in patients with extensively pretreated refractory colorectal cancer metastatic to the liver. Hum Gene Ther. 2010 Sep;21(9):1119–28.
28967558	Geletneky K, Hajda J, Angelova AL, Leuchs B, Capper D, Bartsch AJ, et al. Oncolytic H-1 Parvovirus Shows Safety and Signs of Immunogenic Activity in a First Phase I/IIa Glioblastoma Trial. Mol Ther. 2017 Dec 6;25(12):2620–34.
19572105	Gollamudi R, Ghalib MH, Desai KK, Chaudhary I, Wong B, Einstein M, et al. Intravenous administration of Reolysin, a live replication competent RNA virus is safe in patients with advanced solid tumors. Invest New Drugs. 2010 Oct;28(5):641–9.
34426438	Hajda J, Leuchs B, Angelova AL, Frehtman V, Rommelaere J, Mertens M, et al. Phase 2 Trial of Oncolytic H-1 Parvovirus Therapy Shows Safety and Signs of Immune System Activation in Patients With Metastatic Pancreatic Ductal Adenocarcinoma. Clin Cancer Res. 2021 Oct 15;27(20):5546–56.
20670951	Harrington KJ, Hingorani M, Tanay MA, Hickey J, Bhide SA, Clarke PM, et al. Phase I/II study of oncolytic HSV GM-CSF in combination with radiotherapy and cisplatin in untreated stage III/IV squamous cell cancer of the head and neck. Clin Cancer Res. 2010 Aug 1;16(15):4005–15.
20484020	Harrington KJ, Karapanagiotou EM, Roulstone V, Twigger KR, White CL, Vidal L, et al. Two-stage phase I dose-escalation study of intratumoral reovirus type 3 dearing and palliative radiotherapy in patients with advanced cancers. Clin Cancer Res. 2010 Jun 1;16(11):3067–77.

32669371	Harrington KJ, Kong A, Mach N, Chesney JA, Fernandez BC, Rischin D, et al. Talimogene Laherparepvec and Pembrolizumab in Recurrent or Metastatic Squamous Cell Carcinoma of the Head and Neck (MASTERKEY-232): A Multicenter, Phase 1b Study. Clin Cancer Res. 2020 Oct 1;26(19):5153–61.
15334111	Harrow S, Papanastassiou V, Harland J, Mabbs R, Petty R, Fraser M, et al. HSV1716 injection into the brain adjacent to tumour following surgical resection of high-grade glioma: safety data and long-term survival. Gene Ther. 2004 Nov;11(22):1648–58.
36863095	Hecht JR, Raman SS, Chan A, Kalinsky K, Baurain J-F, Jimenez MM, Garcia MM, Berger MD, Lauer UM, Khattak A, et al. Phase Ib Study of Talimogene Laherparepvec in Combination with Atezolizumab in Patients with Triple Negative Breast Cancer and Colorectal Cancer with Liver Metastases. ESMO Open 2023 Apr ;8(2) :100884.
15961518	Heinzerling L, Künzi V, Oberholzer PA, Kündig T, Naim H, Dummer R. Oncolytic measles virus in cutaneous T-cell lymphomas mounts antitumor immune responses in vivo and targets interferon-resistant tumor cells. Blood. 2005 Oct 1;106(7):2287–94.
25714011	Hemminki O, Parviainen S, Juhila J, Turkki R, Linder N, Lundin J, et al. Immunological data from cancer patients treated with Ad5/3-E2F-Δ24-GMCSF suggests utility for tumor immunotherapy. Oncotarget. 2015 Feb 28;6(6):4467–81.
21427706	Heo J, Breitbach CJ, Moon A, Kim CW, Patt R, Kim MK, et al. Sequential therapy with JX-594, a targeted oncolytic poxvirus, followed by sorafenib in hepatocellular carcinoma: preclinical and clinical demonstration of combination efficacy. Mol Ther. 2011 Jun;19(6):1170–9.
23396206	Heo J, Reid T, Ruo L, Breitbach CJ, Rose S, Bloomston M, et al. Randomized dose-finding clinical trial of oncolytic immunotherapeutic vaccinia JX-594 in liver cancer. Nat Med. 2013 Mar;19(3):329–36.
29801474	Hirooka Y, Kasuya H, Ishikawa T, Kawashima H, Ohno E, Villalobos IB, et al. A Phase I clinical trial of EUS-guided intratumoral injection of the oncolytic virus, HF10 for unresectable locally advanced pancreatic cancer. BMC Cancer. 2018 May 25;18(1):596.
17289893	Hotte SJ, Lorence RM, Hirte HW, Polawski SR, Bamat MK, O'Neil JD, et al. An optimized clinical regimen for the oncolytic virus PV701. Clin Cancer Res. 2007 Feb 1;13(3):977–85.
17121894	Hu JCC, Coffin RS, Davis CJ, Graham NJ, Groves N, Guest PJ, et al. A phase I study of OncoVEXGM-CSF, a second-generation oncolytic herpes simplex virus expressing granulocyte macrophage colony-stimulating factor. Clin Cancer Res. 2006 Nov 15;12(22):6737–47.
28177438	Husseini F, Delord JP, Fournel-Federico C, Guitton J, Erbs P, Homerin M, et al. Vectorized gene therapy of liver tumors: proof-of-concept of TG4023 (MVA-FCU1) in combination with flucytosine. Ann Oncol. 2017 Jan 1;28(1):169–74.
21772252	Hwang TH, Moon A, Burke J, Ribas A, Stephenson J, Breitbach CJ, et al. A mechanistic proof-of-concept clinical trial with JX-594, a targeted multi-mechanistic oncolytic poxvirus, in patients with metastatic melanoma. Mol Ther. 2011 Oct;19(10):1913–22.

33168658	Jiffry J, Thavornwatanayong T, Rao D, Fogel EJ, Saytoo D, Nahata R, et al. Oncolytic Reovirus (pelareorep) Induces Autophagy in KRAS-mutated Colorectal Cancer. Clin Cancer Res. 2021 Feb 1;27(3):865–76.
29653857	Jonker DJ, Tang PA, Kennecke H, Welch SA, Cripps MC, Asmis T, et al. A Randomized Phase II Study of FOLFOX6/Bevacizumab With or Without Pelareorep in Patients With Metastatic Colorectal Cancer: IND.210, a Canadian Cancer Trials Group Trial. Clin Colorectal Cancer. 2018 Sep;17(3):231-239.e7.
34782633	Kai M, Marx AN, Liu DD, Shen Y, Gao H, Reuben JM, et al. A phase II study of talimogene laherparepvec for patients with inoperable locoregional recurrence of breast cancer. Sci Rep. 2021 Nov 15;11(1):22242.
23493351	Kanerva A, Nokisalmi P, Diaconu I, Koski A, Cerullo V, Liikanen I, et al. Antiviral and antitumor T-cell immunity in patients treated with GM-CSF-coding oncolytic adenovirus. Clin Cancer Res. 2013 May 15;19(10):2734–44.
22316603	Karapanagiotou EM, Roulstone V, Twigger K, Ball M, Tanay M, Nutting C, et al. Phase I/II trial of carboplatin and paclitaxel chemotherapy in combination with intravenous oncolytic reovirus in patients with advanced malignancies. Clin Cancer Res. 2012 Apr 1;18(7):2080–9.
26176043	Kasuya H, Kodera Y, Nakao A, Yamamura K, Gewen T, Zhiwen W, et al. Phase I Dose-escalation Clinical Trial of HF10 Oncolytic Herpes Virus in 17 Japanese Patients with Advanced Cancer. Hepatogastroenterology. 2014 May;61(131):599–605.
24484178	Kaufman HL, Kim DW, Kim-Schulze S, DeRaffele G, Jagoda MC, Broucek JR, et al. Results of a randomized phase I gene therapy clinical trial of nononcolytic fowlpox viruses encoding T cell costimulatory molecules. Hum Gene Ther. 2014 May;25(5):452–60.
31971541	Kelly CM, Antonescu CR, Bowler T, Munhoz R, Chi P, Dickson MA, et al. Objective Response Rate Among Patients With Locally Advanced or Metastatic Sarcoma Treated With Talimogene Laherparepvec in Combination With Pembrolizumab: A Phase 2 Clinical Trial. JAMA Oncol. 2020 Mar 1;6(3):402–8.
24553100	Kicielinski KP, Chiocca EA, Yu JS, Gill GM, Coffey M, Markert JM. Phase 1 clinical trial of intratumoral reovirus infusion for the treatment of recurrent malignant gliomas in adults. Mol Ther. 2014 May;22(5):1056–62.
23756180	Kim KH, Dmitriev IP, Saddekni S, Kashentseva EA, Harris RD, Aurigemma R, et al. A phase I clinical trial of Ad5/3-Δ24, a novel serotype-chimeric, infectivity-enhanced, conditionally-replicative adenovirus (CRAd), in patients with recurrent ovarian cancer. Gynecol Oncol. 2013 Sep;130(3):518–24.
23677592	Kim MK, Breitbach CJ, Moon A, Heo J, Lee YK, Cho M, et al. Oncolytic and immunotherapeutic vaccinia induces antibody-mediated complement-dependent cancer cell lysis in humans. Sci Transl Med. 2013 May 15;5(185):185ra63.
20978148	Kimball KJ, Preuss MA, Barnes MN, Wang M, Siegal GP, Wan W, et al. A phase I study of a tropism-modified conditionally replicative adenovirus for recurrent malignant gynecologic diseases. Clin Cancer Res. 2010 Nov 1;16(21):5277–87.
32047956	Kiyohara E, Tanemura A, Nishioka M, Yamada M, Tanaka A, Yokomi A, et al. Intratumoral injection of hemagglutinating virus of Japan-envelope vector yielded an antitumor effect for advanced melanoma: a phase I/IIa clinical study. Cancer Immunol Immunother. 2020 Jun;69(6):1131–40.

25728527	Kolb EA, Sampson V, Stabley D, Walter A, Sol-Church K, Cripe T, et al. A phase I trial and viral clearance study of reovirus (Reolysin) in children with relapsed or refractory extra-cranial solid tumors: a Children's Oncology Group Phase I Consortium report. Pediatr Blood Cancer. 2015 May;62(5):751–8.
20664527	Koski A, Kangasniemi L, Escutenaire S, Pesonen S, Cerullo V, Diaconu I, et al. Treatment of cancer patients with a serotype 5/3 chimeric oncolytic adenovirus expressing GMCSF. Mol Ther. 2010 Oct;18(10):1874–84.
29788332	Kurokawa C, Iankov ID, Anderson SK, Aderca I, Leontovich AA, Maurer MJ, et al. Constitutive Interferon Pathway Activation in Tumors as an Efficacy Determinant Following Oncolytic Virotherapy. J Natl Cancer Inst. 2018 Oct 1;110(10):1123–32.
29432077	Lang FF, Conrad C, Gomez-Manzano C, Yung WKA, Sawaya R, Weinberg JS, et al. Phase I Study of DNX-2401 (Delta-24-RGD) Oncolytic Adenovirus: Replication and Immunotherapeutic Effects in Recurrent Malignant Glioma. J Clin Oncol. 2018 May 10;36(14):1419–27.
29773661	Lauer UM, Schell M, Beil J, Berchtold S, Koppenhöfer U, Glatzle J, et al. Phase I Study of Oncolytic Vaccinia Virus GL-ONC1 in Patients with Peritoneal Carcinomatosis. Clin Cancer Res. 2018 Sep 15;24(18):4388–98.
16638865	Laurie SA, Bell JC, Atkins HL, Roach J, Bamat MK, O'Neil JD, et al. A phase 1 clinical study of intravenous administration of PV701, an oncolytic virus, using two-step desensitization. Clin Cancer Res. 2006 Apr 15;12(8):2555–62.
19092859	Li JL, Liu HL, Zhang XR, Xu JP, Hu WK, Liang M, et al. A phase I trial of intratumoral administration of recombinant oncolytic adenovirus overexpressing HSP70 in advanced solid tumor patients. Gene Ther. 2009 Mar;16(3):376–82.
12632504	Liang W, Wang H, Sun TM, Yao WQ, Chen LL, Jin Y, et al. Application of autologous tumor cell vaccine and NDV vaccine in treatment of tumors of digestive tract. World J Gastroenterol. 2003 Mar;9(3):495–8.
18628758	Liu TC, Hwang T, Park BH, Bell J, Kirn DH. The targeted oncolytic poxvirus JX-594 demonstrates antitumoral, antivascular, and anti-HBV activities in patients with hepatocellular carcinoma. Mol Ther. 2008 Sep;16(9):1637–42.
21106728	Lolkema MP, Arkenau HT, Harrington K, Roxburgh P, Morrison R, Roulstone V, et al. A phase I study of the combination of intravenous reovirus type 3 Dearing and gemcitabine in patients with advanced cancer. Clin Cancer Res. 2011 Feb 1;17(3):581–8.
27031851	Loskog A, Maleka A, Mangsbo S, Svensson E, Lundberg C, Nilsson A, et al. Immunostimulatory AdCD40L gene therapy combined with low-dose cyclophosphamide in metastatic melanoma patients. Br J Cancer. 2016 Apr 12;114(8):872–80.
18615711	Mace ATM, Ganly I, Soutar DS, Brown SM. Potential for efficacy of the oncolytic Herpes simplex virus 1716 in patients with oral squamous cell carcinoma. Head Neck. 2008 Aug;30(8):1045–51.
30691536	Machiels JP, Salazar R, Rottey S, Duran I, Dirix L, Geboes K, et al. A phase 1 dose escalation study of the oncolytic adenovirus enadenotucirev, administered intravenously to patients with epithelial solid tumors (EVOLVE). J Immunother Cancer. 2019 Jan 28;7(1):20.

28289863	Mahalingam D, Fountzilas C, Moseley J, Noronha N, Tran H, Chakrabarty R, et al. A phase II study of REOLYSIN® (pelareorep) in combination with carboplatin and paclitaxel for patients with advanced malignant melanoma. Cancer Chemother Pharmacol. 2017 Apr;79(4):697–703.
29799479	Mahalingam D, Goel S, Aparo S, Patel Arora S, Noronha N, Tran H, et al. A Phase II Study of Pelareorep (REOLYSIN®) in Combination with Gemcitabine for Patients with Advanced Pancreatic Adenocarcinoma. Cancers (Basel). 2018 May 25;10(6):160.
31694832	Mahalingam D, Wilkinson GA, Eng KH, Fields P, Raber P, Moseley JL, et al. Pembrolizumab in Combination with the Oncolytic Virus Pelareorep and Chemotherapy in Patients with Advanced Pancreatic Adenocarcinoma: A Phase Ib Study. Clin Cancer Res. 2020 Jan 1;26(1):71–81
34686353	Manyam M, Stephens AJ, Kennard JA, LeBlanc J, Ahmad S, Kendrick JE, et al. A phase 1b study of intraperitoneal oncolytic viral immunotherapy in platinum-resistant or refractory ovarian cancer. Gynecol Oncol. 2021 Dec;163(3):481–9.
10845725	Markert JM, Medlock MD, Rabkin SD, Gillespie GY, Todo T, Hunter WD, et al. Conditionally replicating herpes simplex virus mutant, G207 for the treatment of malignant glioma: results of a phase I trial. Gene Ther. 2000 May;7(10):867–74.
18957964	Markert JM, Liechty PG, Wang W, Gaston S, Braz E, Karrasch M, et al. Phase Ib trial of mutant herpes simplex virus G207 inoculated pre-and post-tumor resection for recurrent GBM. Mol Ther. 2009 Jan;17(1):199–207.
24572293	Markert JM, Razdan SN, Kuo HC, Cantor A, Knoll A, Karrasch M, et al. A phase 1 trial of oncolytic HSV-1, G207, given in combination with radiation for recurrent GBM demonstrates safety and radiographic responses. Mol Ther. 2014 May;22(5):1048–55.
10505851	Mastrangelo MJ, Maguire HC, Eisenlohr LC, Laughlin CE, Monken CE, McCue PA, et al. Intratumoral recombinant GM-CSF-encoding virus as gene therapy in patients with cutaneous melanoma. Cancer Gene Ther. 1999;6(5):409–22.
28679776	Mell LK, Brumund KT, Daniels GA, Advani SJ, Zakeri K, Wright ME, et al. Phase I Trial of Intravenous Oncolytic Vaccinia Virus (GL-ONC1) with Cisplatin and Radiotherapy in Patients with Locoregionally Advanced Head and Neck Carcinoma. Clin Cancer Res. 2017 Oct 1;23(19):5696–702.
31426803	Minev BR, Lander E, Feller JF, Berman M, Greenwood BM, Minev I, et al. First-in-human study of TK-positive oncolytic vaccinia virus delivered by adipose stromal vascular fraction cells. J Transl Med. 2019 Aug 19;17(1):271.
31413923	Moehler M, Heo J, Lee HC, Tak WY, Chao Y, Paik SW, et al. Vaccinia-based oncolytic immunotherapy Pexastimogene Devacirepvec in patients with advanced hepatocellular carcinoma after sorafenib failure: a randomized multicenter Phase IIb trial (TRAVERSE). Oncoimmunology. 2019;8(8):1615817.
34330766	Monga V, Miller BJ, Tanas M, Boukhar S, Allen B, Anderson C, et al. Intratumoral talimogene laherparepvec injection with concurrent preoperative radiation in patients with locally advanced soft-tissue sarcoma of the trunk and extremities: phase IB/II trial. J Immunother Cancer. 2021 Jul;9(7):e003119.
22886613	Morris DG, Feng X, DiFrancesco LM, Fonseca K, Forsyth PA, Paterson AH, et al. REO-001: A phase I trial of percutaneous intralesional administration of reovirus

	type 3 dearing (Reolysin®) in patients with advanced solid tumors. Invest New Drugs. 2013 Jun;31(3):696–706.
21102422	Nakao A, Kasuya H, Sahin TT, Nomura N, Kanzaki A, Misawa M, et al. A phase I dose-escalation clinical trial of intraoperative direct intratumoral injection of HF10 oncolytic virus in non-resectable patients with advanced pancreatic cancer. Cancer Gene Ther. 2011 Mar;18(3):167–75.
11420638	Nemunaitis J, Cunningham C, Buchanan A, Blackburn A, Edelman G, Maples P, et al. Intravenous infusion of a replication-selective adenovirus (ONYX-015) in cancer patients: safety, feasibility and biological activity. Gene Ther. 2001 May;8(10):746–59.
11103798	Nemunaitis J, Ganly I, Khuri F, Arseneau J, Kuhn J, McCarty T, et al. Selective replication and oncolysis in p53 mutant tumors with ONYX-015, an E1B-55kD gene-deleted adenovirus, in patients with advanced head and neck cancer: a phase II trial. Cancer Res. 2000 Nov 15;60(22):6359–66.
11208818	Nemunaitis J, Khuri F, Ganly I, Arseneau J, Posner M, Vokes E, et al. Phase II trial of intratumoral administration of ONYX-015, a replication-selective adenovirus, in patients with refractory head and neck cancer. J Clin Oncol. 2001 Jan 15;19(2):289–98.
17704755	Nemunaitis J, Senzer N, Sarmiento S, Zhang YA, Arzaga R, Sands B, et al. A phase I trial of intravenous infusion of ONYX-015 and enbrel in solid tumor patients. Cancer Gene Ther. 2007 Nov;14(11):885–93.
19935775	Nemunaitis J, Tong AW, Nemunaitis M, Senzer N, Phadke AP, Bedell C, et al. A phase I study of telomerase-specific replication competent oncolytic adenovirus (telomelysin) for various solid tumors. Mol Ther. 2010 Feb;18(2):429–34.
20501623	Nokisalmi P, Pesonen S, Escutenaire S, Särkioja M, Raki M, Cerullo V, et al. Oncolytic adenovirus ICOVIR-7 in patients with advanced and refractory solid tumors. Clin Cancer Res. 2010 Jun 1;16(11):3035–43.
27039845	Noonan AM, Farren MR, Geyer SM, Huang Y, Tahiri S, Ahn D, et al. Randomized Phase 2 Trial of the Oncolytic Virus Pelareorep (Reolysin) in Upfront Treatment of Metastatic Pancreatic Adenocarcinoma. Mol Ther. 2016 Jun;24(6):1150–8.
32532291	O'Cathail SM, Davis S, Holmes J, Brown R, Fisher K, Seymour L, et al. A phase 1 trial of the safety, tolerability and biological effects of intravenous Enadenotucirev, a novel oncolytic virus, in combination with chemoradiotherapy in locally advanced rectal cancer (CEDAR). Radiat Oncol. 2020 Jun 12;15(1):151.
9816085	Ockert D, Schirrmacher V, Beck N, Stoelben E, Ahlert T, Flechtenmacher J, et al. Newcastle disease virus-infected intact autologous tumor cell vaccine for adjuvant active specific immunotherapy of resected colorectal carcinoma. Clin Cancer Res. 1996 Jan;2(1):21–8.
19565928	Opyrchal M, Aderca I, Galanis E. Phase I clinical trial of locoregional administration of the oncolytic adenovirus ONYX-015 in combination with mitomycin-C, doxorubicin, and cisplatin chemotherapy in patients with advanced sarcomas. Methods Mol Biol. 2009;542:705–17.
28755959	Packiam VT, Lamm DL, Barocas DA, Trainer A, Fand B, Davis RL, et al. An open label, single-arm, phase II multicenter study of the safety and efficacy of

	CG0070 oncolytic vector regimen in patients with BCG-unresponsive non-muscle-invasive bladder cancer: Interim results. Urol Oncol. 2018 Oct;36(10):440–7.
32327728	Packiriswamy N, Upreti D, Zhou Y, Khan R, Miller A, Diaz RM, et al. Oncolytic measles virus therapy enhances tumor antigen-specific T-cell responses in patients with multiple myeloma. Leukemia. 2020 Dec;34(12):3310–22.
11960316	Papanastassiou V, Rampling R, Fraser M, Petty R, Hadley D, Nicoll J, et al. The potential for efficacy of the modified (ICP 34.5(-)) herpes simplex virus HSV1716 following intratumoural injection into human malignant glioma: a proof of principle study. Gene Ther. 2002 Mar;9(6):398–406.
18495536	Park BH, Hwang T, Liu TC, Sze DY, Kim JS, Kwon HC, et al. Use of a targeted oncolytic poxvirus, JX-594, in patients with refractory primary or metastatic liver cancer: a phase I trial. Lancet Oncol. 2008 Jun;9(6):533–42.
26073886	Park SH, Breitbach CJ, Lee J, Park JO, Lim HY, Kang WK, et al. Phase 1b Trial of Biweekly Intravenous Pexa-Vec (JX-594), an Oncolytic and Immunotherapeutic Vaccinia Virus in Colorectal Cancer. Mol Ther. 2015 Sep;23(9):1532–40.
30674657	Pascual-Pasto G, Bazan-Peregrino M, Olaciregui NG, Restrepo-Perdomo CA, Mato-Berciano A, Ottaviani D, et al. Therapeutic targeting of the RB1 pathway in retinoblastoma with the oncolytic adenovirus VCN-01. Sci Transl Med. 2019 Jan 23;11(476):eaat9321.
11980996	Pecora AL, Rizvi N, Cohen GI, Meropol NJ, Sterman D, Marshall JL, et al. Phase I trial of intravenous administration of PV701, an oncolytic virus, in patients with advanced solid cancers. J Clin Oncol. 2002 May 1;20(9):2251–66.
21630267	Pesonen S, Diaconu I, Cerullo V, Escutenaire S, Raki M, Kangasniemi L, et al. Integrin targeted oncolytic adenoviruses Ad5-D24-RGD and Ad5-RGD-D24-GMCSF for treatment of patients with advanced chemotherapy refractory solid tumors. Int J Cancer. 2012 Apr 15;130(8):1937–47.
21556623	Pomer S, Schirrmacher V, Thiele R, Lohrke H, Brkovic D, Staehler G. Tumor response and 4 year survival-data of patients with advanced renal-cell carcinoma treated with autologous tumor vaccine and subcutaneous R-IL-2 and IFN-alpha(2b). Int J Oncol. 1995 May;6(5):947–54.
27298410	Puzanov I, Milhem MM, Minor D, Hamid O, Li A, Chen L, et al. Talimogene Laherparepvec in Combination With Ipilimumab in Previously Untreated, Unresectable Stage IIIB-IV Melanoma. J Clin Oncol. 2016 Aug 1;34(22):2619–26.
26981247	Ranki T, Pesonen S, Hemminki A, Partanen K, Kairemo K, Alanko T, et al. Phase I study with ONCOS-102 for the treatment of solid tumors - an evaluation of clinical response and exploratory analyses of immune markers. J Immunother Cancer. 2016;4:17.
12414631	Reid T, Galanis E, Abbruzzese J, Sze D, Wein LM, Andrews J, et al. Hepatic arterial infusion of a replication-selective oncolytic adenovirus (dl1520): phase II viral, immunologic, and clinical endpoints. Cancer Res. 2002 Nov 1;62(21):6070–9.

15803147	Reid TR, Freeman S, Post L, McCormick F, Sze DY. Effects of Onyx-015 among metastatic colorectal cancer patients that have failed prior treatment with 5-FU/leucovorin. Cancer Gene Ther. 2005 Aug;12(8):673–81.
28886381	Ribas A, Dummer R, Puzanov I, VanderWalde A, Andtbacka RHI, Michielin O, et al. Oncolytic Virotherapy Promotes Intratumoral T Cell Infiltration and Improves Anti-PD-1 Immunotherapy. Cell. 2017 Sep 7;170(6):1109-1119.e10.
24052127	Rojas-Martínez A, Manzanera AG, Sukin SW, Esteban-María J, González-Guerrero JF, Gomez-Guerra L, et al. Intraprostatic distribution and long-term follow-up after AdV-tk immunotherapy as neoadjuvant to surgery in patients with prostate cancer. Cancer Gene Ther. 2013 Nov;20(11):642–9.
25424857	Roulstone V, Khan K, Pandha HS, Rudman S, Coffey M, Gill GM, et al. Phase I trial of cyclophosphamide as an immune modulator for optimizing oncolytic reovirus delivery to solid tumors. Clin Cancer Res. 2015 Mar 15;21(6):1305–12.
32053771	Ruano D, López-Martín JA, Moreno L, Lassaletta Á, Bautista F, Andión M, et al. First-in-Human, First-in-Child Trial of Autologous MSCs Carrying the Oncolytic Virus Icovir-5 in Patients with Advanced Tumors. Mol Ther. 2020 Apr 8;28(4):1033–42.
21304001	Rudin CM, Poirier JT, Senzer NN, Stephenson J, Loesch D, Burroughs KD, et al. Phase I clinical study of Seneca Valley Virus (SVV-001), a replication-competent picornavirus, in advanced solid tumors with neuroendocrine features. Clin Cancer Res. 2011 Feb 15;17(4):888–95.
36669791	Rudin CM, Pandha HS, Zibelman M, Akerley WL, Harrington KJ, Day D, Hill AG, O'Day SJ, Clay TD, Wright GM, et al. Phase 1, Open-Label, Dose-Escalation Study on the Safety, Pharmacokinetics, and Preliminary Efficacy of Intravenous Coxsackievirus A21 (V937), with or without Pembrolizumab, in Patients with Advanced Solid Tumors. J Immunother Cancer. 2023 Jan; 11(1):e005007.
25294913	Sborov DW, Nuovo GJ, Stiff A, Mace T, Lesinski GB, Benson DM, et al. A phase I trial of single-agent reolysin in patients with relapsed multiple myeloma. Clin Cancer Res. 2014 Dec 1;20(23):5946–55.
31605793	Schenk EL, Mandrekar SJ, Dy GK, Aubry MC, Tan AD, Dakhil SR, et al. A Randomized Double-Blind Phase II Study of the Seneca Valley Virus (NTX-010) versus Placebo for Patients with Extensive-Stage SCLC (ES SCLC) Who Were Stable or Responding after at Least Four Cycles of Platinum-Based Chemotherapy: North Central Cancer Treatment Group (Alliance) N0923 Study. J Thorac Oncol. 2020 Jan;15(1):110–9.
1394336	Schlag P, Manasterski M, Gerneth T, Hohenberger P, Dueck M, Herfarth C, et al. Active specific immunotherapy with Newcastle-disease-virus-modified autologous tumor cells following resection of liver metastases in colorectal cancer. First evaluation of clinical response of a phase II-trial. Cancer Immunol Immunother. 1992;35(5):325–30.
18488223	Schulze T, Kemmner W, Weitz J, Wernecke KD, Schirrmacher V, Schlag PM. Efficiency of adjuvant active specific immunization with Newcastle disease virus modified tumor cells in colorectal cancer patients following resection of liver metastases: results of a prospective randomized trial. Cancer Immunol Immunother. 2009 Jan;58(1):61–9.

36113895	Schwarze JK, Tijtgat J, Awada G, Cras L, Vasaturo A, Bagnall C, et al. Intratumoral administration of CD1c (BDCA-1)+ and CD141 (BDCA-3)+ myeloid dendritic cells in combination with talimogene laherparepvec in immune checkpoint blockade refractory advanced melanoma patients: a phase I clinical trial. J Immunother Cancer. 2022 Sep;10(9):e005141.
19884534	Senzer NN, Kaufman HL, Amatruda T, Nemunaitis M, Reid T, Daniels G, et al. Phase II clinical trial of a granulocyte-macrophage colony-stimulating factor-encoding, second-generation oncolytic herpesvirus in patients with unresectable metastatic melanoma. J Clin Oncol. 2009 Dec 1;27(34):5763–71.
34153720	Shirakawa Y, Tazawa H, Tanabe S, Kanaya N, Noma K, Koujima T, et al. Phase I dose-escalation study of endoscopic intratumoral injection of OBP-301 (Telomelysin) with radiotherapy in oesophageal cancer patients unfit for standard treatments. Eur J Cancer. 2021 Aug;153:98–108.
16690359	Small EJ, Carducci MA, Burke JM, Rodriguez R, Fong L, van Ummersen L, et al. A phase I trial of intravenous CG7870, a replication-selective, prostate-specific antigen-targeted oncolytic adenovirus, for the treatment of hormone-refractory, metastatic prostate cancer. Mol Ther. 2006 Jul;14(1):107–17.
33219014	Soliman H, Hogue D, Han H, Mooney B, Costa R, Lee MC, et al. A Phase I Trial of Talimogene Laherparepvec in Combination with Neoadjuvant Chemotherapy for the Treatment of Nonmetastatic Triple-Negative Breast Cancer. Clin Cancer Res. 2021 Feb 15;27(4):1012–8.
36759673	Soliman H, Hogue D, Han H, Mooney B, Costa R, Lee MC, Niell B, Williams A, Chau A, Falcon S, et al. Oncolytic T-VEC Virotherapy plus Neoadjuvant Chemotherapy in Nonmetastatic Triple-Negative Breast Cancer: A Phase 2 Trial. Nat Med. 2023 Feb;29(2):450–457.
15452186	Steiner HH, Bonsanto MM, Beckhove P, Brysch M, Geletneky K, Ahmadi R, et al. Antitumor vaccination of patients with glioblastoma multiforme: a pilot study to assess feasibility, safety, and clinical benefit. J Clin Oncol. 2004 Nov 1;22(21):4272–81.
31570234	Streby KA, Currier MA, Triplet M, Ott K, Dishman DJ, Vaughan MR, et al. First-in-Human Intravenous Seprehvir in Young Cancer Patients: A Phase 1 Clinical Trial. Mol Ther. 2019 Nov 6;27(11):1930–8.
20215509	Thirukkumaran CM, Nodwell MJ, Hirasawa K, Shi ZQ, Diaz R, Luider J, et al. Oncolytic viral therapy for prostate cancer: efficacy of reovirus as a biological therapeutic. Cancer Res. 2010 Mar 15;70(6):2435–44.
35864115	Todo T, Ino Y, Ohtsu H, Shibahara J, Tanaka M. A phase I/II study of triple-mutated oncolytic herpes virus G47Δ in patients with progressive glioblastoma. Nat Commun. 2022 Jul 21;13(1):4119.
35864254	Todo T, Ito H, Ino Y, Ohtsu H, Ota Y, Shibahara J, et al. Intratumoral oncolytic herpes virus G47Δ for residual or recurrent glioblastoma: a phase 2 trial. Nat Med. 2022 Aug;28(8):1630–9.
36271420	Toulmonde M, Cousin S, Kind M, Guegan JP, Bessede A, Le Loarer F, et al. Randomized phase 2 trial of intravenous oncolytic virus JX-594 combined with low-dose cyclophosphamide in patients with advanced soft-tissue sarcoma. J Hematol Oncol. 2022 Oct 21;15(1):149.

35176144	van Putten EHP, Kleijn A, van Beusechem VW, Noske D, Lamers CHJ, de Goede AL, et al. Convection Enhanced Delivery of the Oncolytic Adenovirus Delta24-RGD in Patients with Recurrent GBM: A Phase I Clinical Trial Including Correlative Studies. Clin Cancer Res. 2022 Apr 14;28(8):1572–85.
26862025	van Putten EHP, Wembacher-Schröder E, Smits M, Dirven CMF. Magnetic Resonance Imaging-Based Assessment of Gadolinium-Conjugated Diethylenetriamine Penta-Acetic Acid Test-Infusion in Detecting Dysfunction of Convection-Enhanced Delivery Catheters. World Neurosurg. 2016 May;89:272–9.
18981012	Vidal L, Pandha HS, Yap TA, White CL, Twigger K, Vile RG, et al. A phase I study of intravenous oncolytic reovirus type 3 Dearing in patients with advanced cancer. Clin Cancer Res. 2008 Nov 1;14(21):7127–37.
16285179	Voit C, Kron M, Schwürzer-Voit M, Sterry W. Intradermal injection of Newcastle disease virus-modified autologous melanoma cell lysate and interleukin-2 for adjuvant treatment of melanoma patients with resectable stage III disease. J Dtsch Dermatol Ges. 2003 Feb;1(2):120–5.
26843484	Wheeler LA, Manzanera AG, Bell SD, Cavaliere R, McGregor JM, Grecula JC, et al. Phase II multicenter study of gene-mediated cytotoxic immunotherapy as adjuvant to surgical resection for newly diagnosed malignant glioma. Neuro Oncol. 2016 Aug;18(8):1137–45.
35656636	Yamazaki N, Isei T, Kiyohara Y, Koga H, Kojima T, Takenouchi T, et al. A phase I study of the safety and efficacy of talimogene laherparepvec in Japanese patients with advanced melanoma. Cancer Sci. 2022 Aug;113(8):2798–806.
25292189	Zeh HJ, Downs-Canner S, McCart JA, Guo ZS, Rao UNM, Ramalingam L, et al. First-in-man study of western reserve strain oncolytic vaccinia virus: safety, systemic spread, and antitumor activity. Mol Ther. 2015 Jan;23(1):202–14.
33837053	Zhang B, Huang J, Tang J, Hu S, Luo S, Luo Z, et al. Intratumoral OH2, an oncolytic herpes simplex virus 2, in patients with advanced solid tumors: a multicenter, phase I/II clinical trial. J Immunother Cancer. 2021 Apr;9(4):e002224.
